# Loss of Adenylyl Cyclase 6 in Leptin Receptor‐Expressing Stromal Cells Attenuates Loading‐Induced Endosteal Bone Formation

**DOI:** 10.1002/jbm4.10408

**Published:** 2020-10-14

**Authors:** Mathieu Riffault, Gillian P Johnson, Madeline M Owen, Behzad Javaheri, Andrew A Pitsillides, David A Hoey

**Affiliations:** ^1^ Trinity Centre for Biomedical Engineering Trinity Biomedical Sciences Institute, Trinity College Dublin Dublin Ireland; ^2^ Department of Mechanical, Manufacturing, and Biomedical Engineering School of Engineering, Trinity College Dublin Dublin Ireland; ^3^ Advanced Materials and Bioengineering Research Centre (AMBER) Royal College of Surgeons in Ireland and Trinity College Dublin Dublin Ireland; ^4^ Department of Mechanical, Aeronautical and Biomedical Engineering University of Limerick Limerick Ireland; ^5^ Skeletal Biology Group, Comparative Biomedical Sciences The Royal Veterinary College London United Kingdom

**Keywords:** ADENYLYL CYCLASE 6, BONE ADAPTATION, IN VIVO MECHANICAL LOADING, MECHANOBIOLOGY, STEM CELLS

## Abstract

Bone marrow stromal/stem cells represent a quiescent cell population that replenish the osteoblast bone‐forming cell pool with age and in response to injury, maintaining bone mass and repair. A potent mediator of stromal/stem cell differentiation in vitro and bone formation in vivo is physical loading, yet it still remains unclear whether loading‐induced bone formation requires the osteogenic differentiation of these resident stromal/stem cells. Therefore, in this study, we utilized the leptin receptor (LepR) to identify and trace the contribution of bone marrow stromal cells to mechanoadaptation of bone in vivo. Twelve‐week‐old *Lepr‐cre;tdTomato* mice were subjected to compressive tibia loading with an 11 N peak load for 40 cycles, every other day for 2 weeks. Histological analysis revealed that Lepr‐cre;tdTomato^+^ cells arise perinatally around blood vessels and populate bone surfaces as lining cells or osteoblasts before a percentage undergo osteocytogenesis. Lepr‐cre;tdTomato^+^ stromal cells within the marrow increase in abundance with age, but not following the application of tibial compressive loading. Mechanical loading induces an increase in bone mass and bone formation parameters, yet does not evoke an increase in Lepr‐cre;tdTomato^+^ osteoblasts or osteocytes. To investigate whether adenylyl cyclase‐6 (AC6) in LepR cells contributes to this mechanoadaptive response, *Lepr‐cre;tdTomato* mice were further crossed with AC6^*fl/fl*^ mice to generate a LepR^+^ cell‐specific knockout of *AC6*. These *Lepr‐cre;tdTomato;AC6* ^*fl/fl*^ animals have an attenuated response to compressive tibia loading, characterized by a deficient load‐induced osteogenic response on the endosteal bone surface. This, therefore, shows that Lepr‐cre;tdTomato^+^ cells contribute to short‐term bone mechanoadaptation. © 2020 The Authors. *JBMR Plus* published by Wiley Periodicals LLC on behalf of American Society for Bone and Mineral Research.

## Introduction

Physical loading is a potent regulator of bone anabolism, yet the cellular mechanisms by which this occurs are not fully understood.^(^ ^1^, ^2^ ^)^ This mechanoadaptive response involves bone formation by osteoblasts which are derived from a progenitor or stromal cell population. The finite lifespan of the osteoblast suggests that these cells must be continuously replenished from a progenitor population to meet the cellular demand imposed by mechanical loading; a similar recruitment process operates in response to injury.^(^ ^3^, ^4^, ^5^, ^6^, ^7^ ^)^ Although load‐induced stromal/stem cell differentiation can be indirectly coordinated by the osteocyte,^(^ ^8^ ^)^ a recent study has shown loading‐induced bone formation in a bone explant model that is independent of apparent mechanical stimulation of osteocytes.^(^ ^9^ ^)^ This indicates that applied mechanical stimulation may directly promote bone marrow stem/stromal cells (MSCs) osteogenesis.^(^ ^5^, ^10^ ^)^ However, neither the load‐induced MSC differentiation to osteoblasts nor the mechanistic basis for MSC mechanosensing has been fully elucidated in vivo.

The establishment of a robust MSC marker is critical for their identification and lineage tracing in vivo. MSCs are traditionally described as plastic‐adherent, colony‐forming, non‐hematopoietic cells which can differentiate into chondrogenic, adipogenic and osteogenic progeny.^(^ ^3^, ^11^ ^)^ Furthermore, MSCs are often perivascular in vivo, where murine MSCs are characterized by their lack of expression of hematopoietic (CD45) and endothelial markers (TER‐119) and positive expression of platelet‐derived growth factor receptor alpha (PDGFRα), stem cells antigen‐1 (Sca1), CD51, CD105, CD90, nestin, aSMA, and combinations thereof.^(^ ^3^, ^12^, ^13^, ^14^ ^)^ MSCs can therefore be retrospectively identified based on the above characteristics; yet an appropriate method for their prospective identification is lacking, and hence their location and physiological functions in vivo have remained elusive. Recently, leptin receptor‐positive (LepR^+^) cells were identified as being perivascular and a major source of the stem cell fraction within the bone marrow.^(^ ^3^, ^15^, ^16^, ^17^, ^18^, ^19^ ^)^ Additionally, these LepR^+^ cells were found to express the bone marrow MSC markers PDGFRα and CD51, and to be highly enriched for fibroblast colony‐forming units. Moreover, analyses indicated that LepR^+^ cells in the bone marrow largely overlap with Nestin, an intermediate filament protein that is known as a neural stem/progenitor marker in adult bone marrow.^(^ ^3^, ^20^ ^)^ LepR^+^ cells not only express MSC markers, but have now been shown to function as the main source of new osteoblasts and adipocytes in adult bone marrow and to be recruited to sites of injury to form bony ossicles that support hematopoiesis in vivo.^(^ ^3^ ^)^ Also, osteogenic differentiation of these cells is increased following anabolic stimulation with parathyroid hormone.^(^ ^18^ ^)^ Despite their presence in various tissues and organs^(^ ^21^, ^22^ ^)^ and heterogenous nature,^(^ ^19^ ^)^ these characteristics suggest that LepR^+^ cells are a suitable candidate to determine the role of early progenitors in load‐induced bone anabolic responses.

The candidature of these LepR^+^ cells as a means of prospectively identifying MSC fate is further supported by recent studies highlighting a role for the more committed osteoprogenitors in load‐induced bone formation. Work by Liu and colleagues has focused on the effect of mechanical loading on primitive osteoprogenitors, looking specifically at Prrx1 (paired related homeobox 1) and Sca1‐positive cells,^(^ ^7^ ^)^ and Zannit and Silva investigated the more committed osterix (Osx) positive osteoblast lineage cells.^(^ ^23^ ^)^ Both report proliferation of these Prrx1^+^‐Sca1^+^ and Osx^+^ cells following loading; however, the role of Prrx1 has been predominately characterized on the periosteum.

The osteogenic differentiation of MSCs can be directly driven by mechanical loading in vitro.^(^ ^10^, ^24^ ^)^ Furthermore, we have previously shown that MSCs utilize adenylyl cyclases (ACs) to generate cAMP as a second messenger in this mechanotransduction leading to osteogenesis.^(^ ^25^ ^)^ ACs are a family of transmembrane enzymes that catalyze the cyclization of adenosine triphosphate into cAMP.^(^ ^26^ ^)^ The AC family comprises nine distinct transmembrane isoforms (AC1–AC9), each with individual regulatory properties and restricted expression in only a limited number of tissues.^(^ ^27^, ^28^ ^)^ Specifically, AC6 has been shown to be expressed in skeletal cells and is required for load‐induced bone formation in vivo.^(^ ^29^ ^)^ Interestingly, skeletally mature mice, with a global deletion of AC6, did not present with a skeletal phenotype, but formed significantly less bone than control mice in response to ulna loading, showing that AC6 mediates bone mechanoadaptation.^(^ ^29^ ^)^ Although this study clearly showed a role for AC6 in bone mechanobiology, given the global deletion of this enzyme, the specific cell type and mechanism of action of AC6 in bone mechanoadaptation remains unclear.

The development of the Lepr‐cre mouse model along with specific deletion with Cre‐lox recombination has provided a means to study the fate of these cells and the role of associated molecules. Although LepR+ marrow stromal cells have been shown to be critical to adult bone formation, their role in mechanoadaptation is not known. Therefore, this study aimed to characterize the response of LepR^+^ marrow stromal cells to load‐induced bone formation, and to explore whether these cells or their progeny contribute to load‐related osteogenesis. Utilizing *Lepr‐cre;tdTomato* mice, we have shown that LepR^+^ cells arise perinatally in bone, appearing perivascularly before expanding with age to undergo osteoblastic and osteocytic differentiation and act as the main source of bone‐forming cells. We have shown that loading increases tibial bone formation and has little influence on the percentage of Lepr‐cre;tdTomato^+^ stromal cells within the morrow. Moreover, no significant changes in the percentage of Lepr‐cre;tdTomato^+^ cells lining bone surface or osteocytes were observed, suggesting that loading does not mediate the proliferation or recruitment of LepR^+^ cells. Furthermore, our data show that *AC6* deletion in LepR^+^ cells restricts the endosteal cortical bone response to loading, highlighting the contribution of LepR^+^ cells and a critical role for AC6 in bone mechanoadaptation.

## Materials and Methods

### Mice

All transgenic mice were maintained in a C57BL/6 background. Transgenic mice B6.129‐Lepr^tm2(cre)Rck^/J JAX stock #008320,^(^ ^21^ ^)^ B6.Cg‐Ct(ROSA)26Sor^tm9(CAG‐tdTomato)Hze^/J JAX stock #007909,^(30^ ^)^ and B6;129‐Adcy6^tm1.1Dek^/J JAX stock #022503^(^ ^31^ ^)^ were purchased from the Jackson Laboratory (Bar Harbor, ME, USA), and rederived in‐house. B6.129‐Lepr^tm2(cre)Rck^/J and B6.Cg‐Ct(ROSA)26Sor^tm9(CAG‐tdTomato)Hze^/J were crossed to generate heterozygous B6.129‐Lepr^tm2(cre)Rck^/J and heterozygous B6.Cg‐Ct(ROSA)26Sor^tm9(CAG‐tdTomato)Hze^/J breeding pairs. Female B6.129‐Lepr^tm2(cre)Rck^/J::B6.Cg‐Ct(ROSA)26Sor^tm9(CAG‐tdTomato)Hze^/J offspring heterozygous for B6.Cg‐Ct(ROSA)26Sor^tm9(CAG‐tdTomato)Hze^/J were used for all studies. This *Lepr‐cre;tdTomato* mouse facilitates the labeling of cells actively expressing the leptin receptor, in addition to their progeny irrespective of receptor expression. Heterozygous *Lepr‐cre;tdTomato* mice were subsequently crossed with B6;129‐Adcy6^tm1.1Dek^/J to generate animals with a knockout for *AC6* in Lepr‐cre;tdTomato‐expressing cells resulting in a *Lepr‐cre;tdTomato;AC6* ^*fl/fl*^ mouse. Genotyping was achieved using DNA extracted from the ear and performed by Transnetyx (Memphis, TN, USA). All animals were maintained in groups of four under specific pathogen‐free conditions at 24°C ± 2°C with a 12‐hour light/dark cycle and were provided with water and *ad libitum* diets. The procedures performed in this study were approved by Trinity College Dublin Animal Research Ethics Committee and Health Products Regulatory Authority in Ireland.

### Histological analysis

Embryos, organs, and tibias from all groups were dissected, fixed for 12 hours in neutral buffered formalin (Sigma‐Aldrich, St. Louis, MO, USA), decalcified in 10% EDTA (Sigma‐Aldrich), and processed for standard paraffin embedding. Transverse 10‐μm sections were taken from individual samples and two sections were used in subsequent procedures. Prior to staining, sections were dewaxed and rehydrated. For hematoxylin and eosin (H&E) staining, sections were stained with HARRIS hematoxylin solution (Sigma‐Aldrich) for 4 min before rinsing and staining with eosin Y solution (Sigma‐Aldrich) for 2 min. Sections were subsequently rehydrated and mounted using Distyrene Plasticizer Xylene (DPX) (Sigma‐Aldrich). Slides were imaged on an Aperio Scanscope CS2 (Leica Biosystems, Wetzlar, Germany). For immunofluorescence studies, bone tissue was fixed, decalcified, and cryo‐embedded. Sections of 20 μm were sliced with a cryostat. Then 4,6‐diamidino‐2‐phenylindole (DAPI) at 1:2000 in PBS (Sigma‐Aldrich) was applied to all samples for 5 min prior to sample‐mounting on glass slides using ProLong Gold mounting medium (Invitrogen, Carlsbad, CA, USA). Leptin receptor staining was performed after antigen retrieval with proteinase K solution (20 min at 37°C) in a humidified chamber. Slides were then washed with PBS‐Tween 0.5% v/v and blocked (5% BSA in PBS, 1 hour at 37°C). Slides were incubated in the primary antibody against leptin receptor (1:200; AF497; R&D Systems, Minneapolis, MN, USA), washed, and then further incubated in secondary antibody (1:500; Ab150129; Thermo Fisher Scientific, Waltham, MA, USA). DAPI at 1:2000 in PBS was then applied before mounting using ProLong Gold mounting medium. Imaging was performed on the Leica SP7 (Leica Microsystems, Wetzlar, Germany) scanning confocal microscope at ×20.

### Flow cytometry

To quantify the percentage of Tomato^+^ cells in a given population, organs were harvested, minced, and homogenized, and cell suspension filtered through a 70‐um cell strainer. After centrifugation, cell pellets were resuspended in red blood cell lysis buffer (20mM of Tris, 150mM of NH4Cl in diH20), for 5 min on ice, then washed and resuspended in 1‐mL flow cytometry buffer composed of PBS (Sigma‐Aldrich) with 0.5% BSA (Sigma‐Aldrich) and 2mM EDTA (Sigma‐Aldrich, pH 7.2).

Left and right tibias were isolated, and the bone marrow was flushed from the marrow cavity with 3‐mL DMEM (Sigma‐Aldrich). Once flushed, cells were centrifuged at 400*g* for 5 min and resuspended in 1‐mL red blood cell lysis buffer for 5 min on ice. Cells were washed before subsequent re‐uspension in 2% PBS‐ FBS and incubated on ice for 30 min. Cells were then incubated for 30 min on ice with CD45 (CD45‐BV421, 563890; 1:100; BD Biosciences, San Jose, CA, USA) and TER‐119 (TER‐119‐BB515, 564760; 1:100; BD Biosciences) antibodies. After washing in PBS, cells were resuspended in 1‐mL flow cytometry buffer. Flow cytometry analysis was performed on a BD LSRFortessa (BD Biosciences) at medium speed and gated at 100,000 events of Tomato^+^ cells.

### In vivo axial tibia loading

Mice at 12 weeks of age were initially anesthetized using 4% isoflurane and then maintained at 1.5% to 2% isoflurane during the remainder of the procedure. The right tibia was placed between two cups attached to an electromagnetic loading system with feedback control (ElectroForce 5500; TA Instruments, New Castle, DE, USA ). After an initial 2‐N load, a peak compressive load of 11 N was applied, for 40 cycles with 10 s of rest between each cycle every second day for 2 weeks as previously described.^(^ ^32^ ^)^ Left tibias served as nonloaded internal controls. Body weight was measured at 12 weeks of age and on subsequent loading days. All animals were euthanized on day 18 and prepared for either dynamic histomorphometric, histological, or flow cytometry analysis.

### Microcomputed tomography analysis

Mice were placed under isoflurane‐induced anesthesia as described above. Tibias were imaged by in vivo μCT (Scanco VivaCT 80; Scanco Medical AG, Brüttisellen, Switzerland). The cortical area was scanned with a voxel size of 25 μm. Scans were performed using a voltage of 70 kVp, a current of 114 μA and a 200‐ms integration time. A Gaussian filter (σ = 0.8, support = 1) was used to suppress noise and a global threshold of 150 was applied for analysis or cortical bone scans. The bone volume, cortical area and thickness, second moment of area around major/minor (I_min_ and I_max)_ were quantified using scripts provided by Scanco.

Whole‐body scans were taken for phenotypic analysis of *Lepr‐cre;tdTomato;AC6* ^*fl/fl*^ mice. Briefly, after euthanasia, whole‐body scans were performed at 15‐μm voxel size. Scans were performed using a voltage of 70 kVp, a current of 114 μA, and a 200‐ms integration time. A Gaussian filter (σ = 0.8, support = 1) was used to suppress noise and a global threshold of 150 was applied to generate the 3D reconstruction using scripts provided by Scanco.

Whole‐bone analysis was performed on data sets derived from CT scans using BoneJ^(^ ^33^ ^)^ (version 1.4.2), an ImageJ plugin (NIH, Bethesda, MD, USA; https://imagej.nih.gov/ij/). Following segmentation and removal of fibula from the data set, a minimum bone threshold was selected using a histogram‐based method in ImageJ that utilizes all pixels in a stack to construct a histogram and was further confirmed using ImageJ Threshold function. A threshold of 100 was applied to all data sets to separate higher density bone from soft tissues and air. This threshold was used in Slice Geometry function within BoneJ to calculate bone cross‐sectional area, second moment of area around the minor axis (I_max_), second moment of area around the major axis (I_min_), mean thickness determined by local thickness in two dimensions (cortical thickness), ellipticity and predicted resistance to torsion (J). The most proximal (0%–15%) and the most distal portions (85%–100%) of tibial length were excluded from analysis, as these regions include trabecular bone.

### Dynamic histomorphometry

Mice were injected with calcein (15 mg/kg body weight; Sigma‐Aldrich) on the third and sixth day of loading. Left and right tibias were isolated, cleaned of soft tissue, fixed in formalin (Sigma‐Aldrich). and stored in 70% ethanol for dynamic histomorphometry. The tibias were dehydrated in graded alcohol (70%–100%), infiltrated with three changes of Technovit 9100 methyl methacrylate (C N Technical Services Ltd, Wisbech, England), and embedded in Technovit 9100 following the manufacturer's recommendation. Transverse sections of the embedded tibia midshaft were imaged on a Leica SP7 (Leica Microsystems) scanning confocal microscope. Measurements of the bone perimeter, single‐label perimeter, double‐label perimeter, and double‐label area were completed with Fiji^(^ ^34^ ^)^ (version 1.6.0_24) and used to calculate mineralizing surface/bone surface, mineral apposition rate (MAR), and bone formation rate (BFR)/bone surface. Measurements were taken at both the endosteum and periosteum.

### Immunofluorescence image analysis

A ROI for cortical bone spanning 100 slices (2500 μm) was selected 3 mm from the tibia–fibula junction towards the tibial proximal metaphysis. Using Fiji, the length of the bone surface covered by Tomato^+^ cells at both endosteal and periosteal surfaces, and the number of Tomato^+^ cells embedded within bone were counted within the ROI.

To determine if endosteal regions showing bone formation by dynamic histomorphometry correlate with regions where Tomato^+^ cells are observed on confocal images, both sets of images were analyzed with Fiji. To compare between different animals, the total length of the endosteum was measured and expressed in percentage (0% starting at the tibial ridge, going clockwise to 100%). Locations, where one or two labels of calcein are observed, were determined and plotted against the total length of the endosteum for static and loaded bones. Then, the presence of tdTomato^+^ cells along the endosteum was observed and plotted against the total length of the endosteum. Results were averaged and pooled in clusters of 5%.

### Statistical analysis

For flow cytometry of different tissues, a one‐way ANOVA analysis was performed with Tukey correction. Dynamic histomorphometry analysis of *Lepr‐cre;tdTomato;AC6* ^*fl/fl*^ and comparison with WT mice was performed with a two‐way ANOVA with Tukey correction. For all other studies, unpaired two‐tailed student *t* test with Wilcoxon correction was employed. Data were analyzed using Graph Pad Prism 8 (GraphPad Software, Inc., La Jolla, CA, USA); for gross cortical bone morphology analysis, graphs were plotted using programming language R, version 3.1.3 (R Foundation for Statistical Computing, Vienna, Austria; http://www.r-project.org). The number of animals is detailed in the captions for each figure. In all experiments, *p* < 0.05 was considered statistically significant.

## Results

### LepR ^+^ bone marrow cells are the main source of bone‐forming cells

LepR^+^ bone marrow cells are the main source of bone forming cells on the early endosteal surface and later periosteal surface in addition to embedded osteocytes. We first analyzed the spatiotemporal expression of LepR^+^ cells in our model. Tomato^+^ cells were identified prenatally at E19.5 in the brain and ossification zone of the radius, ulna, and tibia (Fig. S1). The pattern of Tomato^+^ cells was further investigated in all major organs postnatally (Fig. S2). H&E staining was used to investigate the anatomy of organs and to more accurately identify the location of Tomato^+^ cells at 8 and 12 weeks of age (Fig. S2A–F). Tomato^+^ cells were found in various organs including the liver, kidney medulla, lung, spleen, and heart (Fig. S2A,B, D–F). Tomato^+^ cells increased with age, from 8 to 12 weeks, in each of these organs. Quantification of cell number within each organ of the *Lepr‐cre;tdTomato* mouse was performed using flow cytometry at 12 weeks, which further highlighted the spatial differences in Tomato^+^ cells. At 12 weeks of age, Tomato^+^ cells accounted for <7% of cells in each organ, with the exception of the liver where 38% of cells were Tomato^+^.

**Fig 1:**
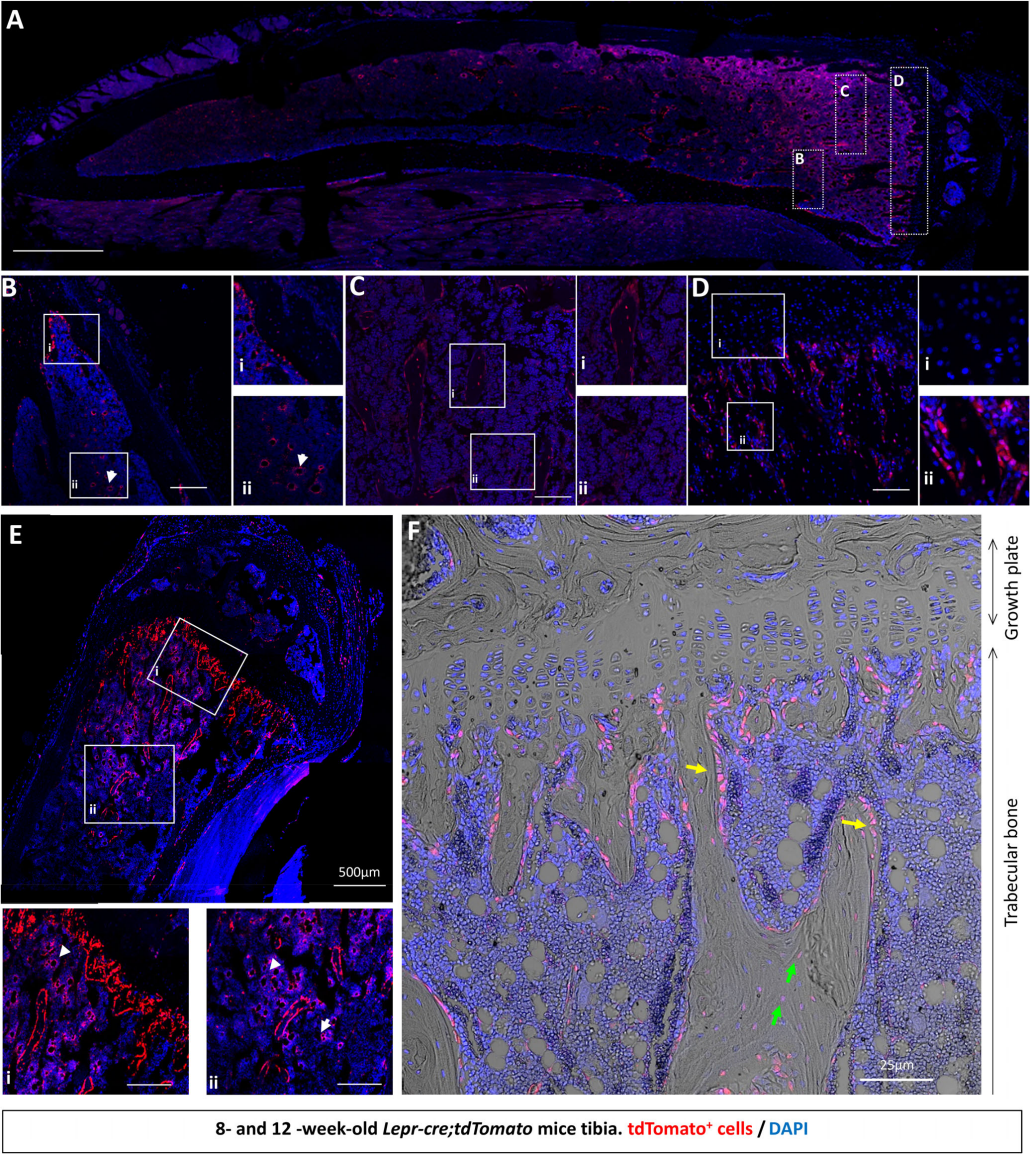
tdTomato^+^ bone marrow cells appear around sinusoids and contribute to osteoblast and osteocyte populations over time in trabecular bone. To assess whether *LepR‐cre* was actively expressed in adult tibia, limbs were harvested from 8‐ and 12‐week‐old *Lepr*‐cre*;tdTomato* mice and processed for histological analyses with the nuclear dye 4,6‐diamidino‐2‐phenylindole (DAPI). (*A*) Representative image of an 8‐week‐old tibia, showing ROIs. (*B*–*D*) Confocal microscopy revealed tdTomato^+^ signal in 8‐week‐old trabecular bone marrow (*B*‐*B*i), perivascularly in the marrow space (arrow head; *B*ii), in trabecular bone (*C*), and below the growth plate (*D*). (*D*i) No staining was found in the growth plate. (*E*–*F*) Confocal microscopy revealed tdTomato^+^ signal in 12‐week‐old mice along the trabecular bone (*E*) and in trabecular bone marrow (*E*i,ii). (*E*i,ii) tdTomato^+^ was found to be perivascular in the marrow space (arrow head). (*F*) No staining was found in the growth plate. Additionally, tdTomato^+^ is expressed on the bone surface (yellow arrow) and embedded within bone (green arrow) in 12‐week‐old mice. *N* = 4. Scale bar = 50 μm unless otherwise indicated.

The expression of Lepr‐cre;tdTomato^+^ cells in 8‐ and 12‐week‐old mice was analyzed in greater detail within the tibia. Sagittal sections of the tibia were imaged using confocal microscopy and the trabecular and cortical bone regions examined for patterns of Tomato^+^ cell expression (Fig. 1). First, investigation of the trabecular region of the tibia of 8‐week‐old *Lepr‐cre;tdTomato* mice, revealed the presence of Tomato^+^ cells within the marrow space between trabeculae (Fig. 1*B*i,*C*ii) where these cells located around sinusoids (Fig. 1*B*ii). Small populations of Tomato^+^ cells were also found lining and embedded within trabecular struts (Fig. 1*B*i,*C*i,*D*ii). Although Tomato^+^ cells are located perivascularly and along the bone surface, no Tomato^+^ cells were found in the growth plate (Fig. 1*D*i). By 12 weeks of age, the prevalence of Tomato^+^ cells located perivascularly within the trabecular bone marrow increased (Fig. 1*E*i,*E*ii), whereas Tomato^+^ cells also increased along and within the trabecular bone. Interestingly, at 12 weeks of age, these cells along the surface of trabecular bone morphologically resembled that of osteoblasts (cuboidal) and bone‐lining cells (flattened) (Fig. 1*F*, yellow arrows) suggestive of osteoblastic differentiation of LepR^+^ bone marrow stromal cells. Furthermore, the population of Tomato^+^ cells embedded within the trabecular bone (Fig. 1*F*, green arrows) is evidence of osteocytic differentiation.

**Fig 2:**
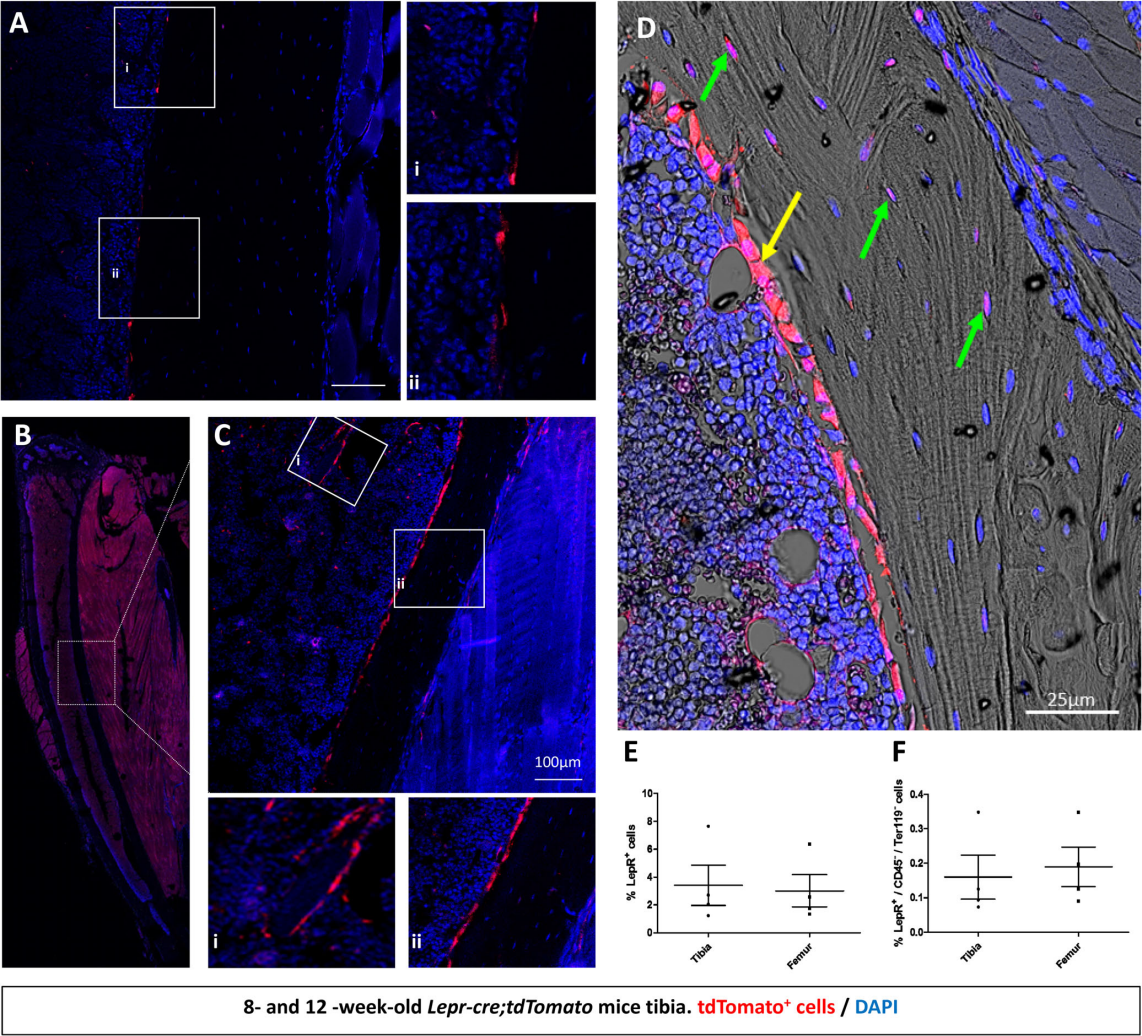
tdTomato^+^ bone marrow cells appear around sinusoids and contribute to osteoblast and osteocyte populations over time in cortical bone. To assess whether *LepR‐cre* was actively expressed in adult tibia, limbs were harvested from 8‐ and 12‐week‐old mice. *Lepr‐cre;tdTomato* mice were processed for histological analyses with the nuclear dye 4,6‐diamidino‐2‐phenylindole (DAPI). (*A*) tdTomato^+^ signal was found on the endosteal surface of cortical bone at 8 weeks. (*B*–*D*) Representative image of a 12‐week‐old tibia. (*C*i,ii) confocal microscopy revealed LepR signal perivascularly in the marrow space (*C*i) and along the cortical bone surface (*C*ii). (*D*) LepR is expressed on the bone surface (yellow arrow) and embedded within bone (green arrow). *N* = 4. Scale bar = 50 μm. (*E*) Flow cytometry analyses revealed that in 12‐week‐old mice tdTomato^+^ make‐up 1.23% to 7.65% and 1.35% to 6.36% of bone marrow cells in the tibia and femur, respectively. (*F*) Exclusion of CD45/Ter119^+^ cells reveals 0.07% to 0.35% and 0.09% to 0.34% tdTomato^+^ cells in the tibia and femur, respectively. *N* = 3. Values are percentages ±SD.

Examining the cortical bone region of the tibial mid‐diaphysis, a small population of Tomato^+^ cells were found perivascularly within the marrow and along the bone surface at 8 weeks of age (Fig. 2*A*). The pattern of expansion of this cell population seen in trabecular bone also held true when the cortical bone was further examined (Fig. 2*B*–*D*); at 12 weeks, Tomato^+^ cells are found perivascularly, along the endosteal surface (Fig. 2*C*ii and *D*, yellow arrow) and embedded within the cortical bone (Fig. 2*D*, green arrows). This observation was confirmed using flow cytometry, which showed that the percentage of Tomato^+^ cells in the marrow is 3.41 ± 2.50% in the tibia and 3.02 ± 1.98% in the femur in 12‐week‐old mice (Fig. 2*E*). Furthermore, LepR^+^CD45^−^Ter119^−^ bone marrow stromal cells accounted for 0.16 ± 0.11% of bone marrow cells within the tibia (Fig. 2*F*). Together, these data suggest that Lepr‐cre;tdTomato^+^ bone marrow stromal cells appear perivascularly, where they expand with age, are recruited to the bone surface of both trabecular and cortical bone, and undergo osteoblastic and osteocytic differentiation.

### Tibial loading enhances endosteal and periosteal cortical bone formation

**Fig 3:**
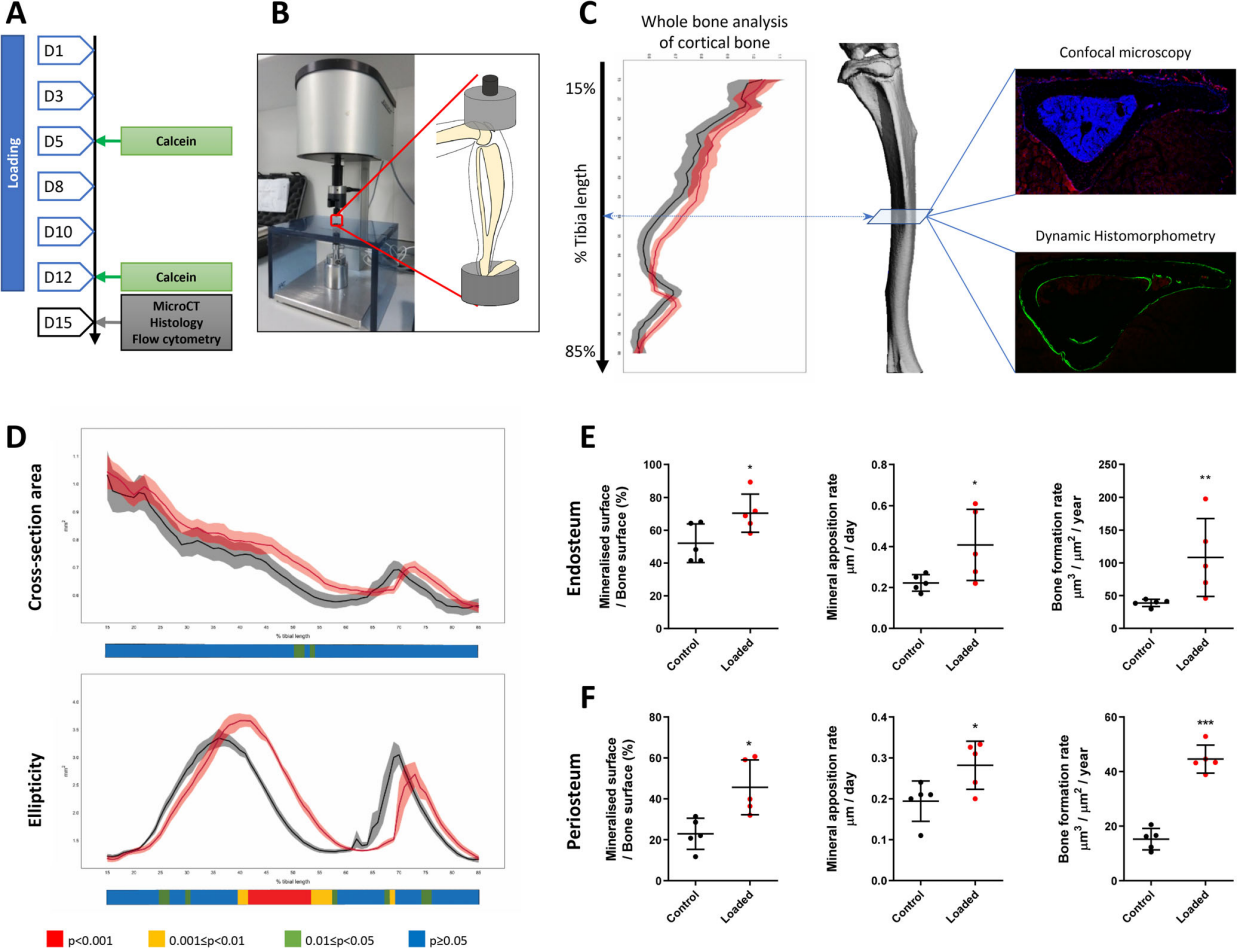
Axial tibia loading of 12‐week‐old *Lepr*‐cre*;tdTomato* mice. (*A*) Schematic of the experimental plan and tibia loading setup. (*B*) The right tibia of 12‐week‐old mice was axially loaded at 11 N for 40 cycles with 10‐second rest periods per day for 14 days. The left tibias were not loaded and were used as nonloaded internal controls. (*C*) Schematic representation of analyses done on tibias. Whole‐bone μCT was performed and cortical bone analyzed between 15% and 90% of the total tibial length. Confocal microscopy of cryosections and dynamic histomorphometry were performed on cross‐sections located between 45% and 50% of the tibial length. (*D*) Whole‐bone analyses of cortical bone between 15% and 85% of the total tibial length, excluding proximal and distal metaphyseal bone, showing cross‐sectional area and ellipticity. Loaded: red, static: black, line graphs represent means ± SEM, *n* = 7. Statistical significance of differences along the entire tibial shaft is represented as a heat map, red *p* < 0.001, yellow 0.001 ≤ *p* < 0.01, green 0.01 ≤ *p* < 0.05, and blue *p* ≥ 0.05. (*E*–*F*) Dynamic histomorphometry analysis of tibial transverse section reveals tibial compressive loading enhances endosteal and periosteal cortical bone formation. Relative mineralizing surface over bone surface, mineral apposition rate, and bone formation rate at the endosteal (*E*) and periosteal (*F*) surface of mechanically loaded tibia. *N* = 5. Mean ± SD.

To investigate whether there are changes in the LepR^+^ stromal cell pool and their progeny during loading‐induced bone formation, a compressive load of 11 N was applied to the tibia of 12‐week‐old female *Lepr‐cre*;*tdTomato* mice (Fig. 3A–C). Consistent with previous studies, our data show that tibia loading in this model leads to an anabolic response in cortical bone of *Lepr‐cre;tdTomato* mice (Fig. 3D–F). Analysis of the entire tibial cortex by μCT reveals an increase in cross‐sectional area following loading, as well as a greater cross‐sectional ellipticity (Fig. 3D). The second moment of inertia around the major (I_min_) and minor (I_max_) axes and the predicted resistance to torsion (J) are also enhanced following the 2 weeks of loading in *Lepr‐cre;tdTomato* mice (Fig. S4).

Bone formation was also measured on both the endosteal and periosteal surfaces using dynamic histomorphometry, where right (loaded) tibias formed significantly more bone than left (nonloaded) tibias (Fig. 3*E*,*F*). After 2 weeks of loading, we found a significant increase in mineralized surface, MAR, and BFR at both the endosteal (Fig. 3*E*) and periosteal surfaces (Fig. 3*F*). Mineralized surface, MAR, and BFR were increased by 30%, 20%, and 79% on the endosteal surface, respectively (Fig. 3*E*), whereas on the periosteal surface mineralized surface, MAR, and BFR increased by 23%, 10%, and 28%, respectively (Fig. 3*F*).

### Tibial loading does not influence the number of LepR ^+^ bone marrow stromal cells or their progeny

**Fig 4:**
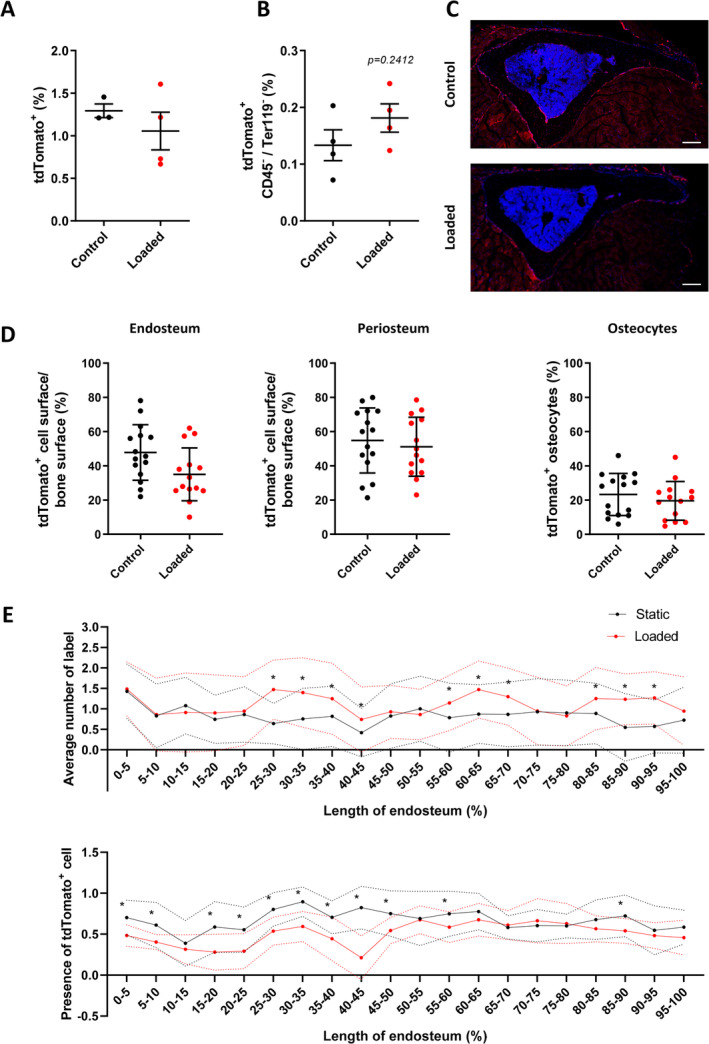
Tibial compressive loading does not alter proliferation or location of Lepr‐cre;tdTomato cells. (*A*–*B*) Flow cytometry analyses of bone marrow cells following mechanical loading of *Lepr*‐cre*;tdTomato* mouse tibia. (*A*) Flow cytometry analyses revealed loading did not alter the percentage of tdTomato^+^ cells. (*B*) Exclusion of CD45 ^+^ and Ter119^+^ cells reveals a trend towards an increase in tdTomato^+^ cells following tibia loading; *n* = 4. (*C*,*D*) Tibial compressive loading does not alter the location of tdTomato^+^ cells. (*C*) Representative image of tibia transection; scale bar = 100 μm. (*D*) The percentage of tdTomato^+^ cells on the endosteal or periosteal surface and embedded with the bone was not altered in cortical bone following tibia compressive loading; *N* = 7. (*E*) Analysis of the location of bone formation along the surface of the endosteum. Upper graph: Average number of label observed by dynamic histomorphometry; *n* = 4. Lower graph: Average number of tdTomato^+^ cells observed lining endosteum surface on confocal images; *n* = 3. Statistical tests employed unpaired two‐tailed student *t* test. Mean ± SD; *p < 0.05.

To determine whether these load‐related increases in cortical bone formation are linked to an expansion and differentiation of the Lepr‐cre;tdTomato^+^ marrow stromal cell population, bone marrow was flushed from the loaded and nonloaded tibias, and flow cytometry was performed to assess the percentage of Tomato^+^ cells. The percentage of Tomato^+^ cells did not increase following tibia loading when no cellular subgroups were excluded (Fig. 4*A*). However, when the CD45^+^ hematopoietic and TER‐119^+^ erythropoietic cells were excluded, the percentage of Tomato^+^ stromal cells was found to be slightly greater (Fig. 4*B*). Although not significant, this could be indicative of a proliferative response in these primitive Tomato^+^ cells. Interestingly, additional staining of LepR (Fig. S3) reveals a colocalization of the signals from tdTomato^+^ and LepR antibody only in the marrow; Tomato^+^ cells lining bone surfaces and Tomato^+^ osteocytes do not show immunolabeling for LepR, indicating that they are not actively expressing LepR at the time of tissue collection on day 17.

The effect of loading on the numbers of Tomato^+^ cells, either lining or embedded within bone, which originated from LepR^+^ stromal cells was furthered assessed using histology (Fig. 4*C*). No change in the percentage of Tomato^+^ cells lining the endosteal or periosteal surface or in the cells embedded in the bone as osteocytes was observed in response to tibial loading (Fig. 4*D*). The location of Tomato^+^ cells lining the endosteal surface was further analyzed and compared with the location where active bone formation had been detected by dynamic histomorphometry (Fig. 4*E*). This revealed that areas of endosteal surface where active bone formation ranged from 25% to 45%, 55% to 70%, and 80% to 95% (Fig. 4*E*, upper graph) failed to exhibit any correlative difference in the local number of Tomato^+^ cells (Fig. 4*E*, lower graph).

These data indicate that our loading protocol, which increases bone formation, does not significantly induce proliferation of LepR^+^ bone marrow stromal cells. Moreover, there is no recruitment of this cell type to the bone surface, suggesting that a reactivation of the cells already present at this location is responsible for the increased load‐related bone accrual response.

### LepR ^+^ cells play a role in loading‐induced bone formation via an AC6‐dependent mechanism

**Fig 5:**
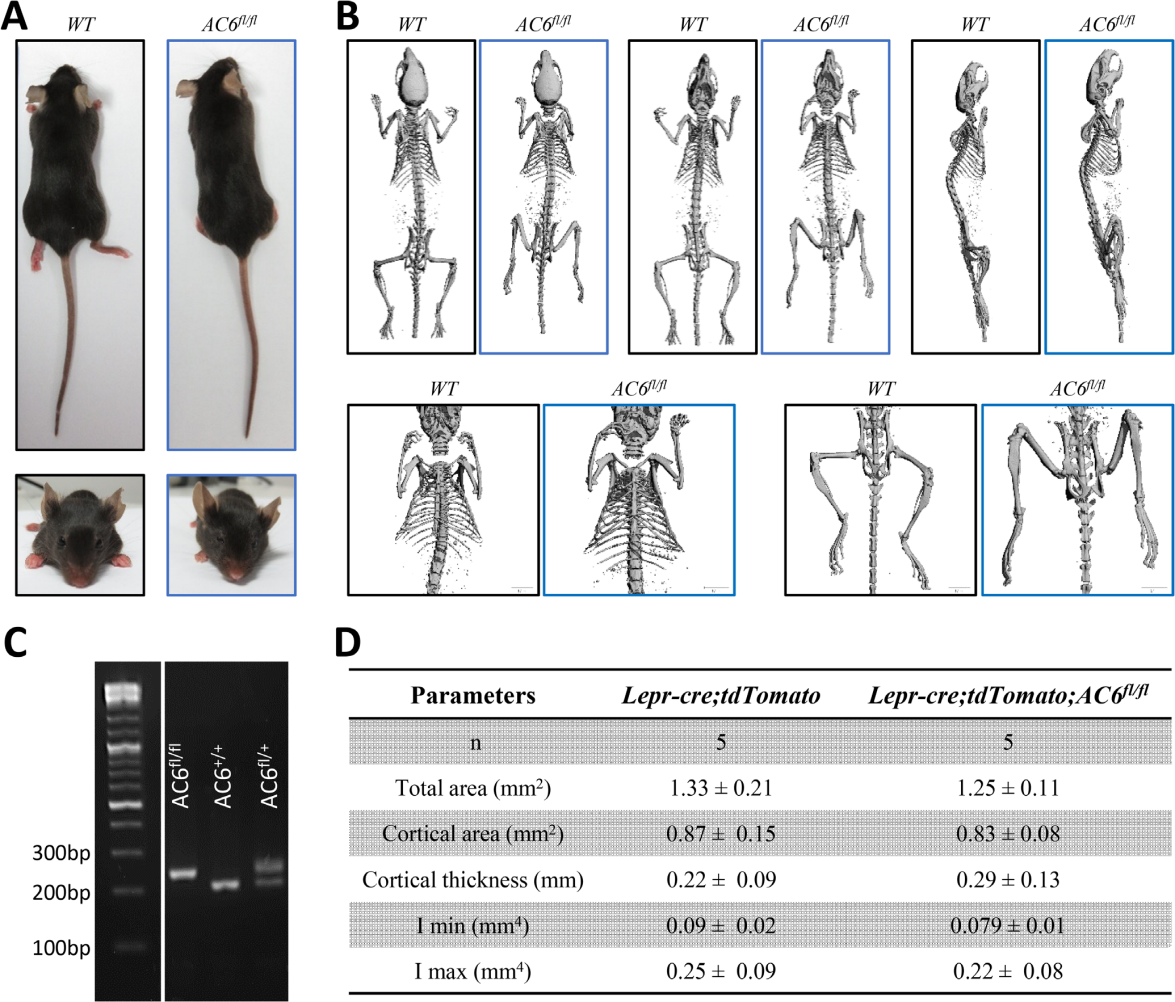
Phenotypic analysis of *Lepr‐cre;tdTomato* and *Lepr‐cre;tdTomato;AC6* ^*fl/fl*^ mice at 8 and 12 weeks. (*A*) Photographs of *Lepr‐cre;tdTomato* and *Lepr‐cre;tdTomato;AC6* ^*fl/fl*^ mice at 12 weeks old. (*B*) Full‐body μCT scans comparing the two genotypes. (*C*) Gel electrophoresis of genotyping showing a band at 260 bp for *AC6* floxed gene. (*D*) Cortical bone midshaft geometry of 12‐week‐old *Lepr‐cre;tdTomato* and *Lepr‐cre;tdTomato;AC6* ^*fl/fl*^ mice.

To investigate whether cells derived from LepR^+^ stromal cells play a role in load‐induced bone formation, we crossed the *Lepr‐cre*;*tdTomato* mouse with the AC6 floxed animal *AC6* ^*fl/fl*^ to generate an *AC6* knockout in leptin receptor‐expressing cells and their progeny (*Lepr‐cre*;*tdTomato;AC6* ^*fl/fl*^). Utilizing a global deletion of AC6, it has been previously shown that AC6 is required for loading‐induced bone formation.^(^ ^29^ ^)^ However, it is unclear in which cell type AC6 is mediating this response. *Lepr‐cre*;*tdTomato*;*AC6* ^*fl/fl*^ mice were healthy and fertile, and appeared phenotypically normal (Fig. 5*A*,*B*, Fig. S5). Body weight of all mice in the study increased with age, with no differences observed between *Lepr‐cre*;*tdTomato* control animals and *Lepr‐cre;tdTomato;AC6* ^*fl/fl*^ mice at any time point (Fig. S5A). On average, the body weights of *Lepr‐cre;tdTomato* and *Lepr‐cre*;*tdTomato;AC6* ^*fl/fl*^ mice were not significantly different at 8 or 12 weeks of age: *Lepr‐cre*;*tdTomato* mice weighed 17.0 ± 0.1 g and 18.9 ± 0.4 g at 8 and 12 weeks, respectively, whereas *Lepr‐cre;tdTomato;AC6* ^*fl/fl*^ mice weighed 17.7 ± 0.4 g and 19.0 ± 0.8 g at 8 and 12 weeks, respectively (Fig. S5A). In addition, μCT analysis was conducted to further examine cortical bone microarchitecture of *Lepr‐cre;tdTomato;AC6* ^*fl/fl*^ and *Lepr‐cre;tdTomato* mice tibias. The total area, cortical area, cortical thickness, I_min_, and I_max_ at the tibial midshaft of *Lepr‐cre;tdTomato;AC6* ^*fl/fl*^ mice were not significantly different from *Lepr‐cre;tdTomato* mice (Fig. 5*C*). Collectively, these data indicate that there were no differences in the skeletal morphology of young‐adult *Lepr‐cre;tdTomato* and *Lepr‐cre;tdTomato;AC6* ^*fl/fl*^ mice. Thus, these results suggest *Lepr‐cre*;*tdTomato;AC6* ^*fl/fl*^ mice do not exhibit a gross morphological or skeletal phenotype, which is consistent with the *AC6* global deletion model.^(^ ^29^ ^)^

**Fig 6:**
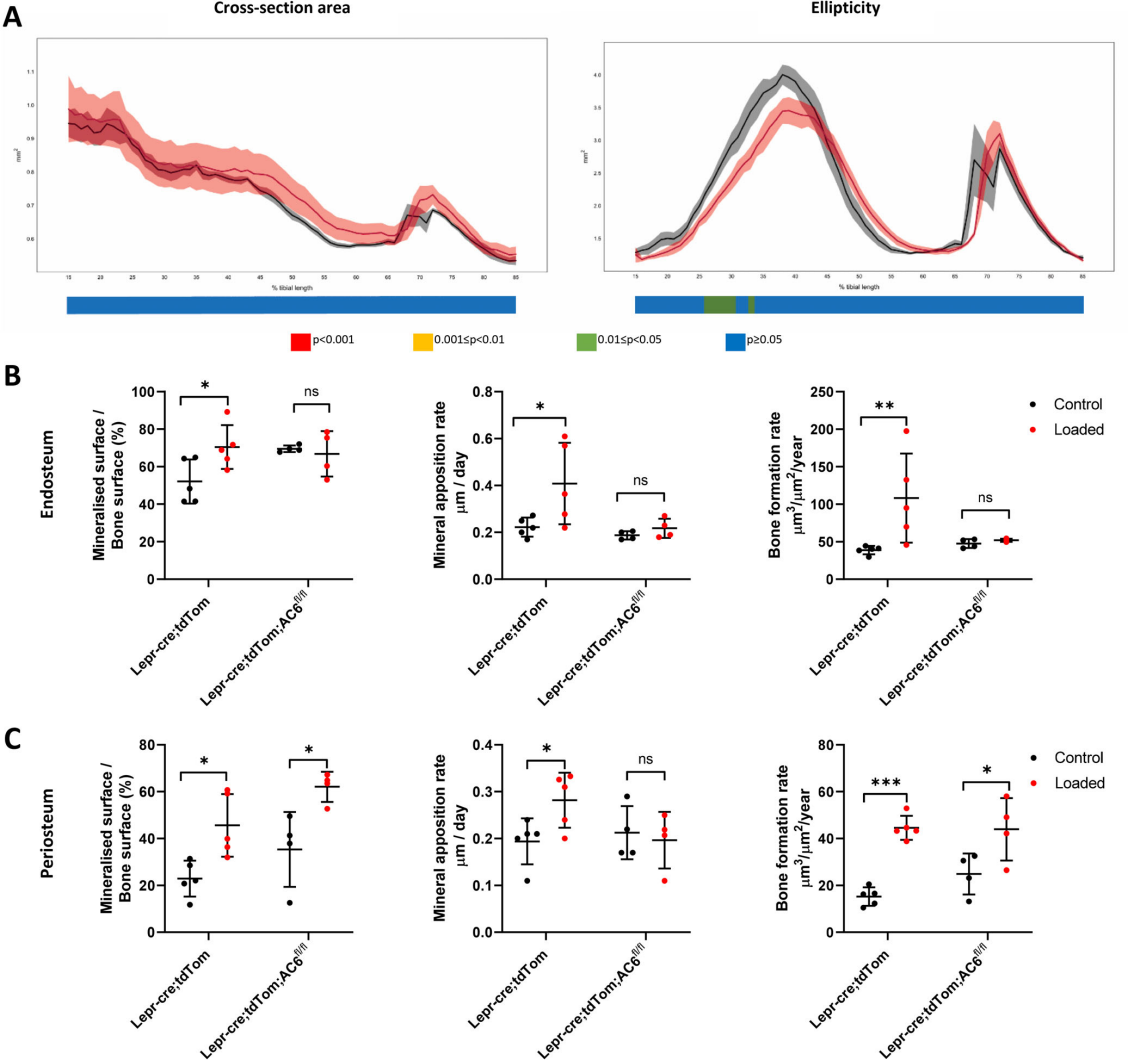
Axial tibia loading of 12‐week‐old *Lepr‐cre;tdTomato;AC6* ^*fl/fl*^ mice. (*A*) Whole‐bone analyses of cortical bone of mice lacking AC6 between 15% and 85% of the total tibial length, excluding proximal and distal methaphyseal bone showing cross‐sectional area and ellipticity. Loaded: red, static: black, line graphs represent means ± SEM, *n* = 6. Statistical significance of differences along the entire tibial shaft is represented as a heat map, red *p* < 0.001, yellow 0.001 ≤ *p* < 0.01, green 0.01 ≤ *p* < 0.05, and blue *p* ≥ 0.05. (*B*,*C*) Mice lacking AC6 showed poor mineralization on the endosteal surface, indicated by a lack of labeling at the endosteal surface in both loaded and nonloaded tibias. (*B*) Relative mineralizing surface over bone surface, mineral apposition rate, and bone formation rate at the endosteal surface of mechanically loaded tibia. (*C*) Relative mineralizing surface over bone surface, mineral apposition rate, and bone formation rate at the periosteal surface. *N* = 5 for *Lepr‐cre;tdTomato*. *N* = 3 for *Lepr‐cre;tdTomato;AC6* ^*fl/fl*^ . Mean ± SD.

As there is no skeletal phenotype following *AC6* deletion, an identical tibial‐loading regime was applied to the *Lepr‐cre;tdTomato;AC6* ^*fl/fl*^ mice, and μCT measurements were taken along the entire tibia length at the end of the loading period (Fig. 6*A*). Interestingly, no changes were observed for tibial cross‐sectional area, ellipticity (Fig. 6*A*), thickness of the cortical bone, I_min_, I_max_ or the resistance to torsion (Fig. S7) following the application of load in these *Lepr‐cre;tdTomato;AC6* ^*fl/fl*^ mice. This result shows that the deletion of *AC6* in a LepR‐specific manner prevents the load‐induced cortical bone formation otherwise observed.

Dynamic histomorphometry was utilized to further evaluate the effect of loading on cortical bone formation in *Lepr‐cre;tdTomato;AC6* ^*fl/fl*^ mice (Fig. 6*B*,*C*). No changes in mineralized surface, MAR, or BFR were found at the endosteal surface of tibial cortical bone in *Lepr‐cre*;*tdTomato;AC6* ^*fl/fl*^ postloading (Fig. 6*B*), which is in agreement with the μCT analysis. However, on the periosteal surface, loading of *Lepr‐cre*;*tdTomato;AC6* ^*fl/fl*^ tibia resulted in an increase in mineralized surface and BFR, whereas no change in MAR was detected (Fig. 6*C*).

**Fig 7:**
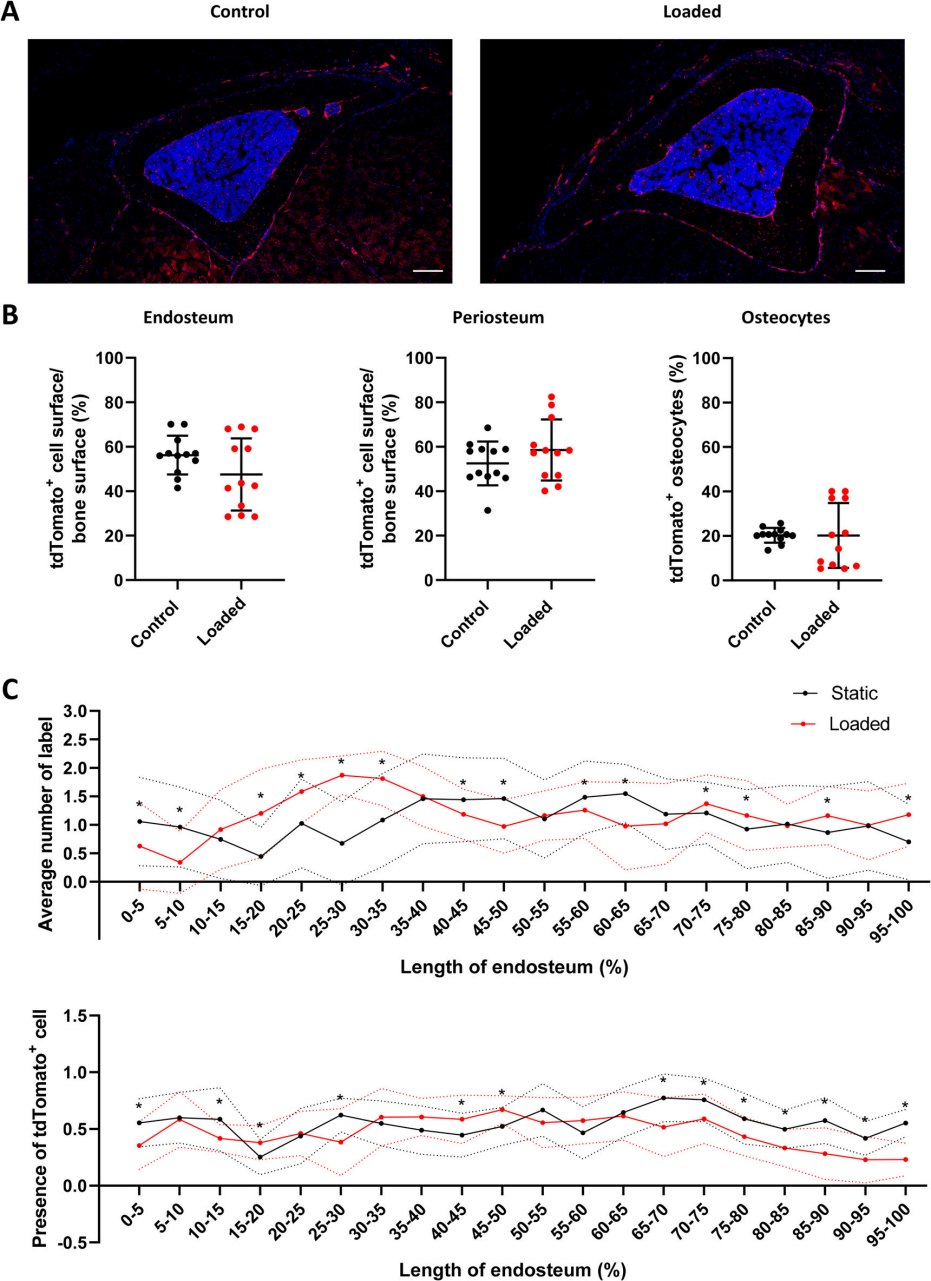
Tibial compressive loading does not alter proliferation or location of Lepr‐cre;tdTomato cells in *Lepr‐cre;tdTomato* and *Lepr‐cre;tdTomato;AC6* ^*fl/fl*^ mice. (*A*) Representative image of tibia transection of *Lepr‐cre;tdTomato;AC6* ^*fl/fl*^ mice following tibia compressive loading; scale bar = 100 μm. (*B*) The percentage of tdTomato^+^ cells on the endosteal and periosteal surfaces and embedded with the bone was not altered in cortical bone; *N* = 4. Statistical tests employed unpaired two‐tailed student *t* test with Wilcoxon correction. Mean ± SD. (*C*) Analysis of the location of bone formation along the surface of the endosteum. Upper graph: Average number of label observed by dynamic histomorphometry; *n* = 4. Lower graph: Average number of tdTomato^+^ cells observed lining endosteum surface on confocal images; *n* = 3). Statistical tests employed unpaired two‐tailed student *t* test. Mean ± SD; *p < 0.05.

This effect of loading on Tomato^+^;AC6^−/−^ cells lining and embedded within bone was further assessed using histology (Fig. 7*A*). Mechanical loading did not change the percentage of Tomato^+^;AC6^−/−^ cells observed in any region of the tibia (Fig. 7*B*). The percentages of Tomato^+^; AC6^−/−^ cells on the endosteum, periosteum, and embedded within the cortical bone were investigated, and no effect of loading on cell number was evident. These data indicate that *Lepr‐cre;tdTomato;AC6* ^*fl/fl*^ animals have both an attenuated endosteal osteogenic response to loading and exhibit no change in the percentage of local Lepr‐cre;tdTomato^+^ cells. This, therefore, indicates that LepR^+^ cells contribute to bone formation on the endosteal surface and that AC6 is required in these cells to mediate this response.

## Discussion

Bone marrow stromal/stem cells represent a quiescent cell population that supply bone‐forming osteoblast cells to maintain tissue homeostasis and to facilitate repair in response to injury. A potent mediator of stromal/stem cell differentiation in vitro and bone formation in vivo is mechanical loading, yet it is unclear whether load‐induced bone formation requires the recruitment and differentiation of resident progenitor cells. Therefore, in this study, we utilized the leptin receptor to identify and trace the contribution of bone marrow stromal cells and their progeny to bone mechanoadaptation. Lepr‐cre;tdTomato^+^ cells were tracked from E19.5 to early adulthood, to find that Lepr‐cre;tdTomato^+^ cells initially appear perivascularly within the marrow, perinatally, and increase in number with age, contributing to osteoblast and osteocyte populations signifying osteogenic lineage commitment. Compressive loading of *Lepr‐cre;tdTomato* tibias resulted in increased bone formation on the endosteal and periosteal surface of cortical bone. Interestingly, no significant increase in the percentage of Lepr‐cre;tdTomato^+^ stromal cells within the bone marrow was observed, whereas no significant changes in the number of Lepr‐cre;tdTomato^+^ cells lining the bone surface or osteocytes embedded in bone were found following loading. *AC6* deletion in Lepr‐cre;tdTomato^+^ cells resulted in a reduced endocortical bone‐forming response to loading, indicating a critical role for Lepr‐cre;tdTomato^+^ cell progeny in loading‐induced bone formation. In summary, these data indicate that mechanical loading does not result in the proliferation of Lepr‐cre;tdTomato^+^ stromal cells within the marrow or in the recruitment of these cells to the bone surface, suggesting that these cells may play either a supportive role in osteogenesis via cell nonautonomous effects, or alternatively Lepr+ cells already present along the bone surface are reactivated, mediating short‐term load‐induced bone formation in a manner that is dependent on AC6.

The leptin receptor is expressed prenatally in bone and brain tissue and becomes widely expressed in nearly all major organs postnatally. Using confocal microscopy, the expression pattern of Lepr‐cre;tdTomato^+^ cells was analyzed in E19.5 mice. During this late stage of gestation, a limited number of Lepr‐cre;tdTomato^+^ cells were found to be present only in the brain and bone tissue. This is consistent with previous work that found no Lepr‐cre;tdTomato^+^ cells in the ossification center of bone at E15.5,^(^ ^20^ ^)^ and limited LepR‐positive cells at E19.5, indicating little contribution of these cells to bone formation at these earlier stages of development.^(^ ^3^ ^)^ The number of Lepr‐cre;tdTomato^+^ in the metaphyseal bone marrow showed a sharp increase by postnatal day P0.5,^(^ ^3^ ^)^ and in 1‐week‐old mice LepR^+^ cells were present throughout the bone marrow.^(^ ^20^ ^)^ Our data, in combination with previous work, suggest that Lepr‐cre;tdTomato^+^ cells increase in the bone marrow during bone maturation. We have also shown that Lepr‐cre;tdTomato^+^ cells were present within the brain at E19.5 and are found in the heart, lungs, spleen, liver, and the medulla region of the kidney in 8‐ and 12‐week‐old animals. Further interrogation by mRNA expression analysis of *LepR* in various mouse tissues also found that the heart and spleen have the lowest expression of LepR of the tissues analyzed,^(^ ^22^ ^)^ which is consistent with our findings. This wide expression of leptin receptor has considerable implications for the use of the leptin receptor for the study of MSC behavior in bone, particularly when combined with Cre‐lox strategy for gene deletion.

Within bone, Lepr‐cre;tdTomato^+^ cells appear perivascularly in the marrow, where they are recruited to the bone surface and commit to the osteogenic lineage with age. The percentage of Lepr‐cre;tdTomato^+^ cells within the tibial marrow increased between 8 and 12 weeks of age, suggesting a maturation‐related expansion of this cell type. This increase in marrow Lepr‐cre;tdTomato^+^ cells was mirrored by an increase in tdTomato^+^ cells on both bone surfaces and embedded with bone. Similar findings were reported by Zhou and colleagues, where the percentage of Lepr‐cre;tdTomato^+^ cells making up *Col2.3‐*GFP^+^ osteoblast cells increased from 10% to 81% from 6 to 14 months of age.^(^ ^3^ ^)^ This earlier study also found that the increase was not caused by the induced expression of LepR at this age, but rather by the proliferation and differentiation of LepR cells resident in the bone marrow.^(^ ^3^ ^)^ Furthermore, we did not observe LepR immunolabeling in cells located on the bone surface or embedded in the bone matrix in our *Lepr‐cre;tdTomato* mice, and studies at 15 weeks in the same mouse model have shown that tdTomato^+^ cells in the bone tissue were osteocalcin‐ and dentin matrix protein‐1– (DMP1‐) expressing mature osteoblasts and osteocytes, respectively.^(^ ^20^ ^)^ Importantly, LepR mRNA was not detectable by quantitative real‐time PCR in the osteoblasts, suggesting that Lepr‐cre;tdTomato^+^ mature bone cells do not autonomously express LepR, but are descendants of LepR^+^ precursors.^(^ ^20^ ^)^ Taken together, these data show that the leptin receptor is a robust marker of MSCs in vivo to trace their progeny.

Although the contribution of Lepr‐cre;tdTomato^+^ cells to adult bone formation has been investigated, their contribution to load‐induced bone formation has not been examined to date. Herein, in vivo mechanical loading of *Lepr‐cre;tdTomato* mouse tibia resulted in no change in the percentage of Lepr‐cre;tdTomato^+^ cells along the bone surface or osteocytes, suggesting that this loading protocol does not initiate recruitment of Lepr‐cre;tdTomato^+^ marrow cells, but instead activates resident cells at the bone surface. This is in close agreement with several previous observations made in other models of bone loading, where early‐loading–related activation of osteoblast metabolic activity was observed and where there was evidence for the direct transformation from quiescence to bone formation in the adult periosteum following a single brief period of bone loading.^(^ ^35^, ^36^ ^)^

The loading protocol used in this study spanned 2 weeks; therefore, although loading induced a trend in an increase in the percentage of Lepr‐cre;tdTomato^+^ marrow stromal cells, these LepR^+^ progenitor cells did not contribute to bone formation within the time frame studied. Recent work from Yang and colleagues also found a lack of response in this cell population following 10 days of iPTH treatment in the femoral marrow.^(^ ^18^ ^)^ Interestingly, this finding of reactivation of mature cells is consistent with a study by Chow and colleagues, where loading of the caudal vertebra resulted in reactivation of previously quiescent bone‐lining cells.^(^ ^37^ ^)^ As with the present study, the rapidity with which new bone was formed following mechanical stimulation raised the potential for this bone formation to occur via the reactivation of cells already along the bone surface, rather than recruitment from the stem cell niche. Recently, Matic and colleagues observed labeled bone surface cells at time points extending beyond the reported lifespan for an osteoblast, suggesting that continuous reactivation of bone‐lining cells is a potential mechanism of adult bone adaptation.^(^ ^38^ ^)^ Other recent studies have reported proliferation of osteoprogenitor Prrx1^+^‐Sca1^+^ cells^(^ ^7^ ^)^ and preosteoblast Osx^+^ cells^(23)^ as a major contributor to loading‐induced bone formation and not the differentiation of stem cells, which further strengthens our findings.

The specific knockout of *AC6* in LepR^+^ cells does not induce a skeletal phenotype, but results in abolition of load‐induced adaptive responses at the endocortical surface, providing a critical role for LepR^+^ cells and their progeny in bone mechanoadaptation. The absence of a basal skeletal phenotype in *Lepr‐cre;tdTomato;AC6* ^*fl/fl*^ mice suggests that AC6 does not play a role in skeletal development. However, the disruption of bone mechanoadaptation on the endosteal surface in *Lepr‐cre;tdTomato;AC6* ^*fl/fl*^ mice proves the importance of AC6 in load‐induced bone formation. At the time of loading, approximately 50% of the bone surface is covered by cells derived from LepR^+^ cells; these cells may be responsible for the anabolic bone response—this is consistent with our in vitro studies highlighting a vital role for AC6 in MSC and mature bone cell mechanotransduction.^(^ ^25^, ^29^ ^)^ However, we cannot yet directly rule out the possibility that LepR^+^ cells in the marrow may contribute to the actvation of non‐Lepr‐cre;tdTomato^+^ cells on the bone surface in a nonautonomous manner.

Although the response on the periosteal surface was blunted, the bone‐forming response observed at this location may be attributed to other non‐LepR^+^ cells potentially recruited from the periosteum.^(^ ^39^ ^)^ For example, Duchamp de Lageneste and colleagues described a population of skeletal stem cells labeled by Prrx1^+^ in the periosteum that expressed markers shown to define mouse skeletal stem cells, but were negative for leptin receptor.^(^ ^39^ ^)^ Moreover, it was shown by Moore and colleagues that Prrx1^+^ cells resident in the periosteum can sense and respond to physical stimulation in vivo and contribute to the load‐induced bone formation.^(^ ^40^ ^)^ Additional work is required to draw conclusive findings; however, in our experiment this LepR^−^/Prrx1^+^ cell population would not have been targeted by the AC6 deletion, and thus may play a role in the load‐induced bone‐forming response observed on the periosteal surface.

This diminished mechanoadaptive response is in agreement with work examining a global knockout of *AC6*, where AC6 deletion resulted in an inhibited response to ulna loading,^(^ ^29^ ^)^ and further strengthens the potential involvement of the primary cilium, to which AC6 localizes, in bone mechanoadaptation.^(^ ^41^, ^42^ ^)^ Furthermore, as with the *Lepr‐cre;tdTomato* mouse, no change was found in the percentage of Lepr‐cre;tdTomato^+^ cells on the bone surface or embedded within the bone. The lack of bone formation and the failure of loading to induce migration of LepR^+^ cells from the marrow to the bone surface in *Lepr‐cre;tdTomato;AC6* ^*fl/fl*^ mice are consistent with our hypothesis that loading‐induced bone formation occurs via Lepr‐cre;tdTomato^+^ cells, and that this process requires AC6.

## Conclusions

In conclusion, this study has characterized the contribution of LepR^+^ bone marrow stromal cells to bone formation during growth and in response to mechanical loading. Interestingly, although LepR^+^ stromal cells are the main source of osteoblasts and osteocytes with age, they are not recruited to the bone surface in response to short‐term loading. Rather, LepR^+^ cells contribute to bone formation either through a supportive role via cell nonautonomous effects, or alternatively, LepR^+^ cells already present along the bone surface are reactivated. Interestingly, this activation requires AC6, which has previously been shown to be an important component of stem cell and mature bone cell mechanotransduction.

## Disclosures

The authors have no conflicts of interest.

## Author Contributions

**Mathieu Riffault:** Conceptualization; data curation; formal analysis; investigation; methodology; validation; writing‐original draft; writing‐review and editing. **Gillian Johnson:** Conceptualization; data curation; formal analysis; funding acquisition; investigation; methodology; validation; visualization; writing‐original draft; writing‐review and editing. **Madeline Owen:** Data curation; formal analysis. **Behzad Javaheri:** Formal analysis; investigation; methodology; software; validation; writing‐original draft; writing‐review and editing. **Andrew Pitsillides:** Formal analysis; funding acquisition; investigation; methodology; supervision; validation; writing‐original draft; writing‐review and editing.

### Peer Review

The peer review history for this article is available at https://publons.com/publon/10.1002/jbm4.10408.

## Supporting information


**Fig. S1.** tdTomato+ cells arise pre‐natally in numerous tissues including bone. (A‐K) To assess whether Lepr‐cre;tdTomato was actively expressed in embryo tissues, heterozygous embryos were harvested at E19.5 days and processed for histological analyses with Hematoxylin and Eosin staining (A‐B;L‐M) and the nuclear dye DAPI (C‐K; N‐P). (A‐B) Hematoxylin and Eosin staining of an embryo in the sagittal plane (A) and embryo head in the transverse plane (B). (C‐K) Confocal microscopy revealed tdTomato+ staining in the cerebral cortex (C), midbrain (D), cervical spine (E), ribs (F), cerebellum (I), choroid plexus (J) and nasal bone (K). No staining was found in the liver (G) or the intestines (H). (L‐M) Hematoxylin and Eosin staining of embryo fore‐ (L) and hind limbs (M). (N‐P) Confocal microscopy revealed tdTomato+ staining in the ossification zone regions of the radius (N), ulna (O), and tibia (P). n = 4. Scale bar 100 μm
Fig. S2: tdTomato+ cells expand overtime and are present in all major organs. To assess whether Lepr‐cre;tdTomato was actively expressed in adult tissues, organs were harvested from 8 and 12‐week‐old Lepr‐cre;tdTomato mice and processed for histological analyses with the nuclear dye DAPI. (A‐D) Confocal microscopy revealed tdTomato+ signal in various organs including the liver (A), kidney medulla (B), lung (D), spleen (E), and heart (F). tdTomato+ signal is reduced from the medulla to the kidney cortex (C). n = 3. Scale bar 100 μm. (G) Flow cytometry analyses revealed that in 12‐week‐old mice tdTomato+ cells vary between organs, where tdTomato+ expression is lowest in the spleen and highest in the liver. Statistical tests employed was a one‐way ANOVA with Tukey post‐hoc. n = 5. Values are percentages ± SEM.
Fig. S3: Staining with LepR antibody reveals that tdTomato+ cells located along the bone surface and osteocytes do not express leptin receptor. Limbs were harvested from 14 week‐old mice following bone mechanical loading and processed for histological analysis and leptin receptor immunolabeling. Upper panel: static tibia, lower panel: loaded tibia. Nuclei are labeled with DAPI (blue), tdTomato+ cells appear red, and cells labeled with leptin receptor antibody in green. Scale bar 100 μm, zooms i & ii 50 μm.
Fig. S4: Axial tibia loading of 12‐week‐old Lepr‐cre;tdTomato+ mice. Whole bone analyses of cortical bone between 15% and 85% of the total tibial length, excluding proximal and distal methaphyseal bone showing Imin, Imax, mean thickness of the cortex and the resistance to torsion. Loaded: red, static: black, Line graph represent means ± SEM, n = 7. Statistical significance of differences along the entire tibial shaft is represented as a heat map, red p < 0.001, yellow 0.001 ≤ p < 0.01, green 0.01 ≤ p < 0.05 and blue p ≥ 0.05.
Fig. S5: Ablation of AC6 in LepR+ cells does not result in a phenotype. (A) Mean weight progression of both genotypes from 8 to 12 weeks. (B) Summary of phenotypes found in Lepr‐cre;tdTomato and Lepr‐cre;tdTomato;AC6fl/fl mice.
Fig. S6: Patterns of LepR expression in adult Lepr‐cre;tdTomato and Lepr‐cre;tdTomato;AC6fl/fl mice. To assess whether tdTomato+ expression differs with the addition of an Adenylyl cyclase 6 knockdown in adult tissues. Organs were harvested from 14‐week‐old mice and processed for histological analyses and stained with hematoxylin and eosin or labeled with the nuclear dye DAPI. Scale bar 100 μm.
Fig. S7: Axial tibia loading of 12‐week‐old Lepr‐cre;tdTomato+;AC6fl/fl mice. Whole bone analyses of cortical bone between 15% and 90% of the total tibial length, excluding proximal and distal methaphyseal bone showing Imin, Imax, mean thickness of the cortex and the resistance to torsion. Loaded: red, static: black, Line graph represent means ± SEM. Statistical significance of differences along the entire tibial shaft is represented as a heat map, red p < 0.001, yellow 0.001 ≤ p < 0.01, green 0.01 ≤ p < 0.05 and blue p ≥ 0.05.
