# Supplementary material for: Loss of Adenylyl Cyclase 6 in Leptin Receptor‐Expressing Stromal Cells Attenuates Loading‐Induced Endosteal Bone Formation
Source: JBMR Plus. 2020 Oct 14;4(11):e10408. doi: 10.1002/jbm4.10408 (PMC7657397; doi:10.1002/jbm4.10408)
Supplement: Supplementary file 1 — Fig. S1. tdTomato+ cells arise pre‐natally in numerous tissues including bone. (A‐K) To assess whether Lepr‐cre;tdTomato was actively expressed in embryo tissues, heterozygous embryos were harvested at E19.5 days and processed for histological analyses with Hematoxylin and Eosin staining (A‐B;L‐M) and the nuclear dye DAPI (C‐K; N‐P). (A‐B) Hematoxylin and Eosin staining of an embryo in the sagittal plane (A) and embryo head in the transverse plane (B). (C‐K) Confocal microscopy revealed tdTomato+ staining in the cerebral cortex (C), midbrain (D), cervical spine (E), ribs (F), cerebellum (I), choroid plexus (J) and nasal bone (K). No staining was found in the liver (G) or the intestines (H). (L‐M) Hematoxylin and Eosin staining of embryo fore‐ (L) and hind limbs (M). (N‐P) Confocal microscopy revealed tdTomato+ staining in the ossification zone regions of the radius (N), ulna (O), and tibia (P). n = 4. Scale bar 100 μm Fig. S2: tdTomato+ cells expand overtime and are present in all major organs. To assess whether Lepr‐cre;tdTomato was actively expressed in adult tissues, organs were harvested from 8 and 12‐week‐old Lepr‐cre;tdTomato mice and processed for histological analyses with the nuclear dye DAPI. (A‐D) Confocal microscopy revealed tdTomato+ signal in various organs including the liver (A), kidney medulla (B), lung (D), spleen (E), and heart (F). tdTomato+ signal is reduced from the medulla to the kidney cortex (C). n = 3. Scale bar 100 μm. (G) Flow cytometry analyses revealed that in 12‐week‐old mice tdTomato+ cells vary between organs, where tdTomato+ expression is lowest in the spleen and highest in the liver. Statistical tests employed was a one‐way ANOVA with Tukey post‐hoc. n = 5. Values are percentages ± SEM. Fig. S3: Staining with LepR antibody reveals that tdTomato+ cells located along the bone surface and osteocytes do not express leptin receptor. Limbs were harvested from 14 week‐old mice following bone mechanical loading and processed for histologic [file JBM4-4-e10408-s001.docx]

Supplementary information for

**Loss of adenylyl cyclase 6 in leptin receptor expressing stromal cells attenuates loading induced endosteal bone formation**

Mathieu Riffault^1,2,3,*^, Gillian P. Johnson^1,2,3,4,*^, Madeline M. Owen^1,2^, Behzad Javaheri^5^, Andrew A. Pitsillides^5^, David A. Hoey^1,2,3,4^

* Both authors contributed equally to the work

^1^ Trinity Centre for Biomedical Engineering, Trinity Biomedical Sciences Institute, Trinity College Dublin, Dublin, Ireland

^2^ Department of Mechanical, Manufacturing, and Biomedical Engineering, School of Engineering, Trinity College Dublin, Dublin, Ireland

^3^ Advanced Materials and Bioengineering Research Centre (AMBER), Royal College of Surgeons in Ireland and Trinity College Dublin, Dublin, Ireland

^4^ Department of Mechanical, Aeronautical and Biomedical Engineering, University of Limerick, Limerick, Ireland

^5^ Skeletal Biology Group, Comparative Biomedical Sciences, The Royal Veterinary College, London, UK

Running title: Adenylyl Cyclase 6 is required for load-induced bone formation

Corresponding author

David A. Hoey, PhD

Email: dahoey@tcd.ie

**
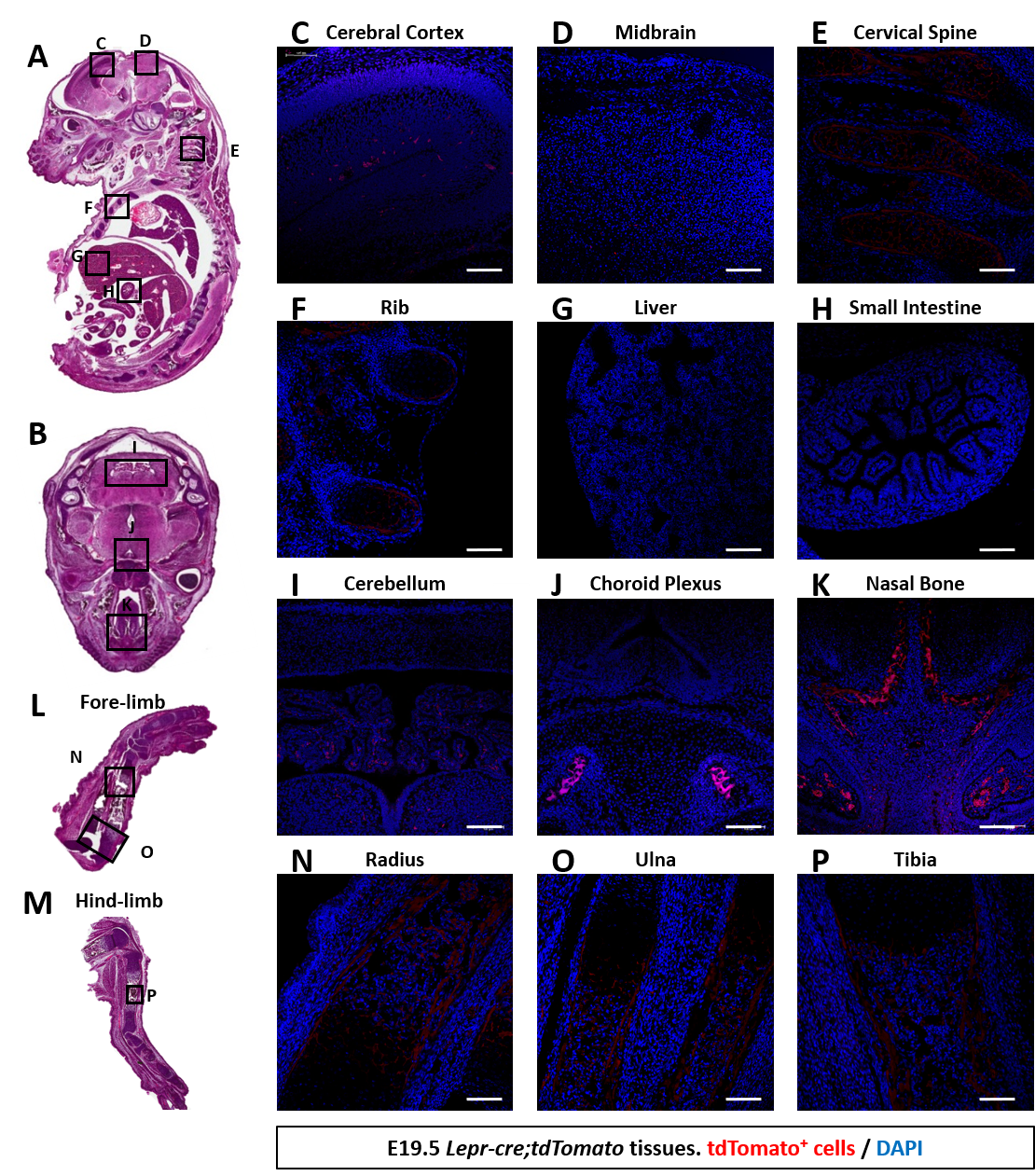
**

**Figure S1:** tdTomato^+^ cells arise pre-natally in numerous tissues including bone. **(A-K)** To assess whether *Lepr-cre;tdTomato* was actively expressed in embryo tissues, heterozygous embryos were harvested at E19.5 days and processed for histological analyses with Hematoxylin and Eosin staining **(A-B;L-M)** and the nuclear dye DAPI **(C-K; N-P)**. **(A-B)** Hematoxylin and Eosin staining of an embryo in the sagittal plane **(A)** and embryo head in the transverse plane **(B)**. **(C-K)** Confocal microscopy revealed tdTomato^+^ staining in the cerebral cortex **(C)**, midbrain **(D)**, cervical spine **(E)**, ribs **(F)**, cerebellum **(I)**, choroid plexus **(J)** and nasal bone **(K)**. No staining was found in the liver **(G)** or the intestines **(H)**. **(L-M)** Hematoxylin and Eosin staining of embryo fore- **(L)** and hind limbs **(M)**. **(N-P)** Confocal microscopy revealed tdTomato^+^ staining in the ossification zone regions of the radius **(N)**, ulna **(O)**, and tibia **(P)**. n=4. Scale bar 100µm

**
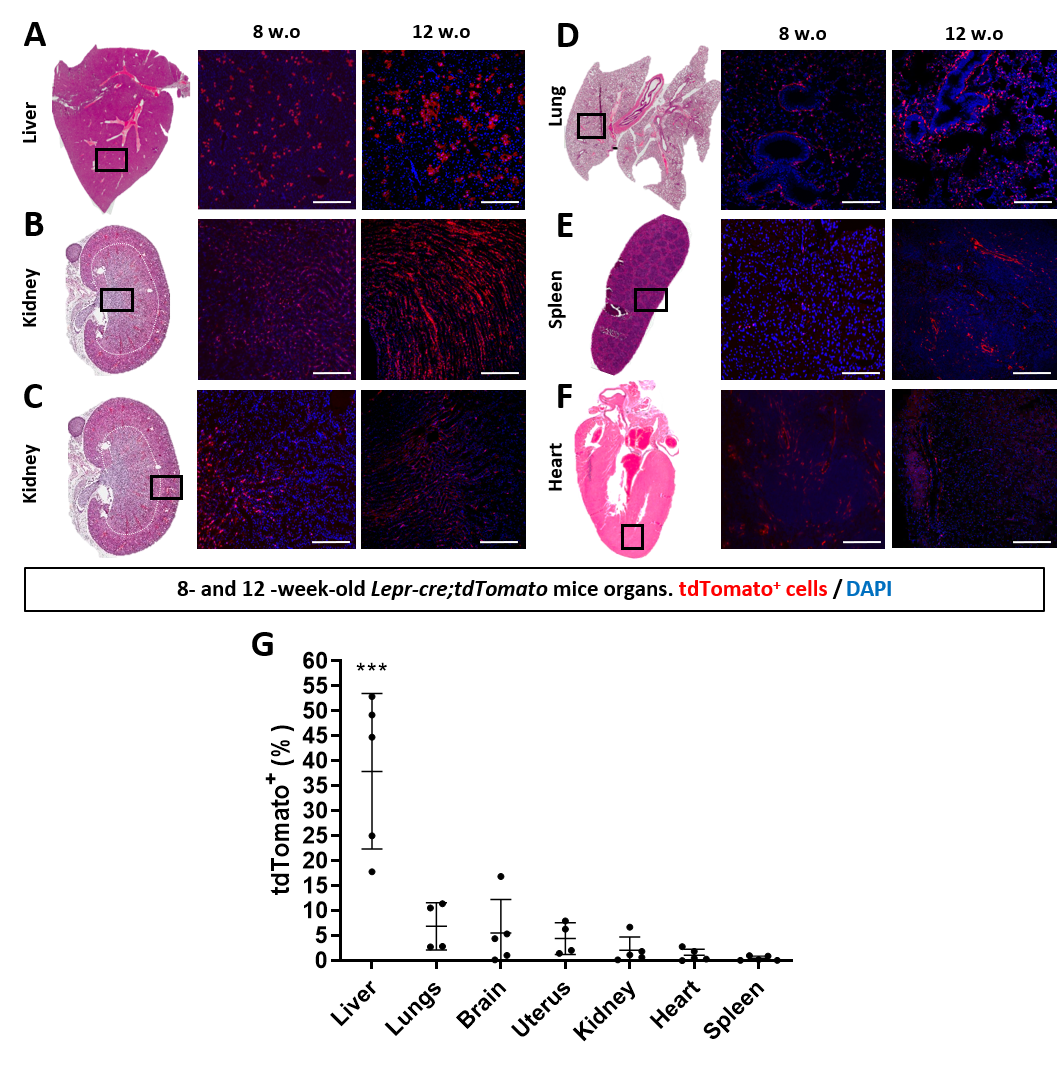
**

**Figure S2:** tdTomato^+^ cells expand overtime and are present in all major organs.To assess whether *Lepr-cre;tdTomato* was actively expressed in adult tissues, organs were harvested from 8 and 12-week-old *Lepr-cre;tdTomato* mice and processed for histological analyses with the nuclear dye DAPI. **(A-D)** Confocal microscopy revealed tdTomato^+^ signal in various organs including the liver **(A)**, kidney medulla **(B)**, lung **(D)**, spleen **(E)**, and heart **(F)**. tdTomato^+^ signal is reduced from the medulla to the kidney cortex **(C)**. n=3. Scale bar 100µm. **(G)** Flow cytometry analyses revealed that in 12-week-old mice tdTomato^+^ cells vary between organs, where tdTomato^+^ expression is lowest in the spleen and highest in the liver. Statistical tests employed was a one-way ANOVA with Tukey post-hoc. n=5. Values are percentages ± SEM

**
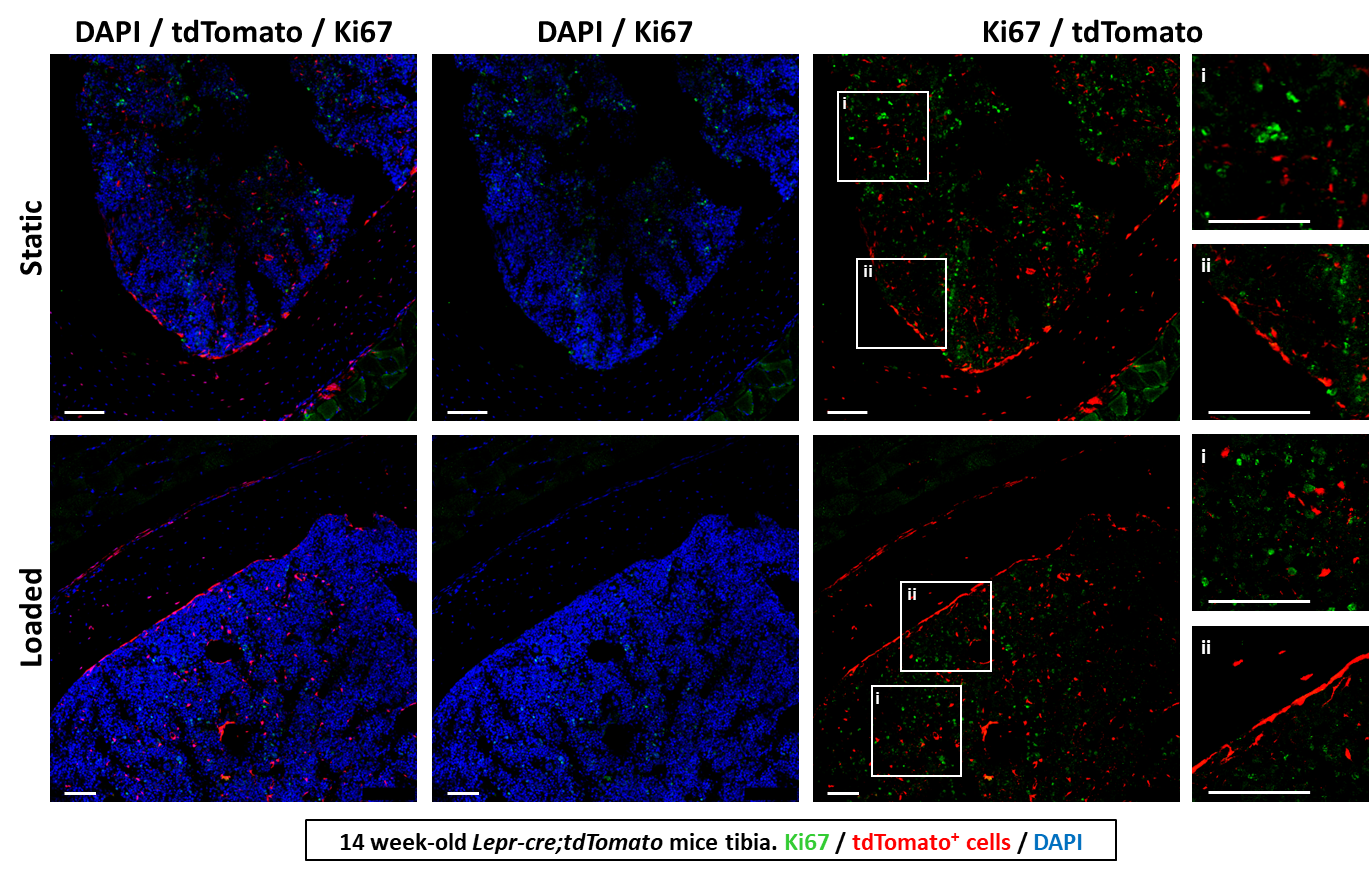
**

**Figure S3:** Staining with LepR antibody reveals that tdTomato+ cells located along the bone surface and osteocytes do not express leptin receptor. Limbs were harvested from 14 week-old mice following bone mechanical loading and processed for histological analysis and Leptin receptor immunolabelling. Upper panel: static tibia, lower panel: loaded tibia. Nuclei are labelled with DAPI (blue), tdTomato+ cells appear red, and cells labelled with leptin receptor antibody in green. Scale bar 100µm, zooms i & ii 50µm.

**
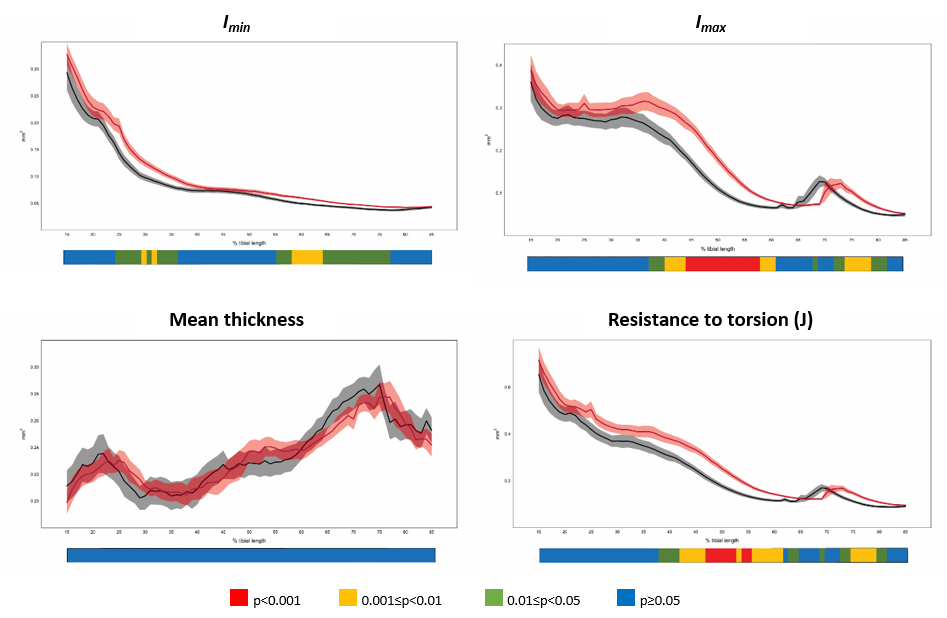
**

**Figure S4:** Axial tibia loading of 12-week-old *Lepr*-cre ;tdTomato^+^ mice. Whole bone analyses of cortical bone between 15-85% of the total tibial length, excluding proximal and distal methaphyseal bone showing I_min_, I_max_, mean thickness of the cortex and the resistance to torsion. Loaded: red, static: black, Line graph represent means +/-SEM, n=7. Statistical significance of differences along the entire tibial shaft is represented as a heat map, red p< 0.001, yellow 0.001≤p<0.01, green 0.01≤p<0.05 and blue p≥0.05.

**
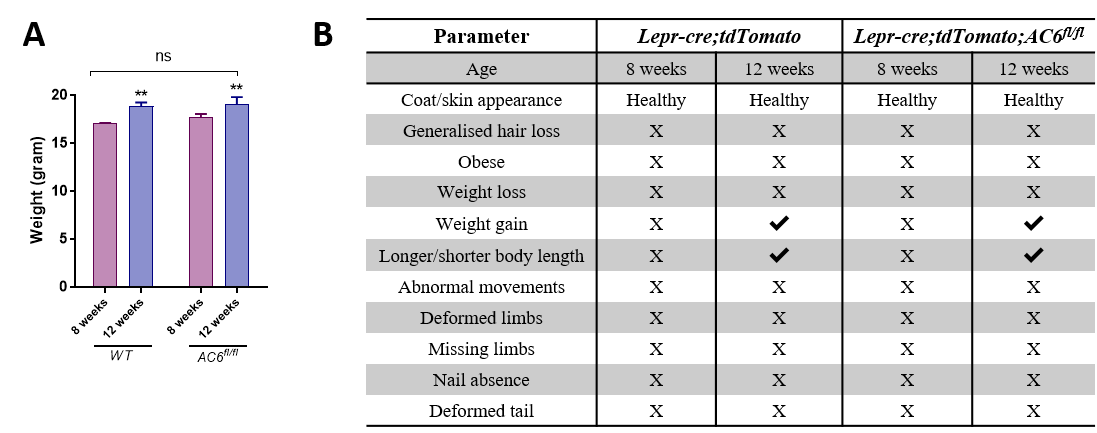
**

**Figure S5:** Ablation of AC6 in LepR+ cells does not result in a phenotype. **(A)** Mean weight progression of both genotypes from 8 to 12 weeks. **(B)** Summary of phenotypes found in *Lepr-cre;tdTomato* and *Lepr-cre;tdTomato;AC6^fl/fl^* mice.

**
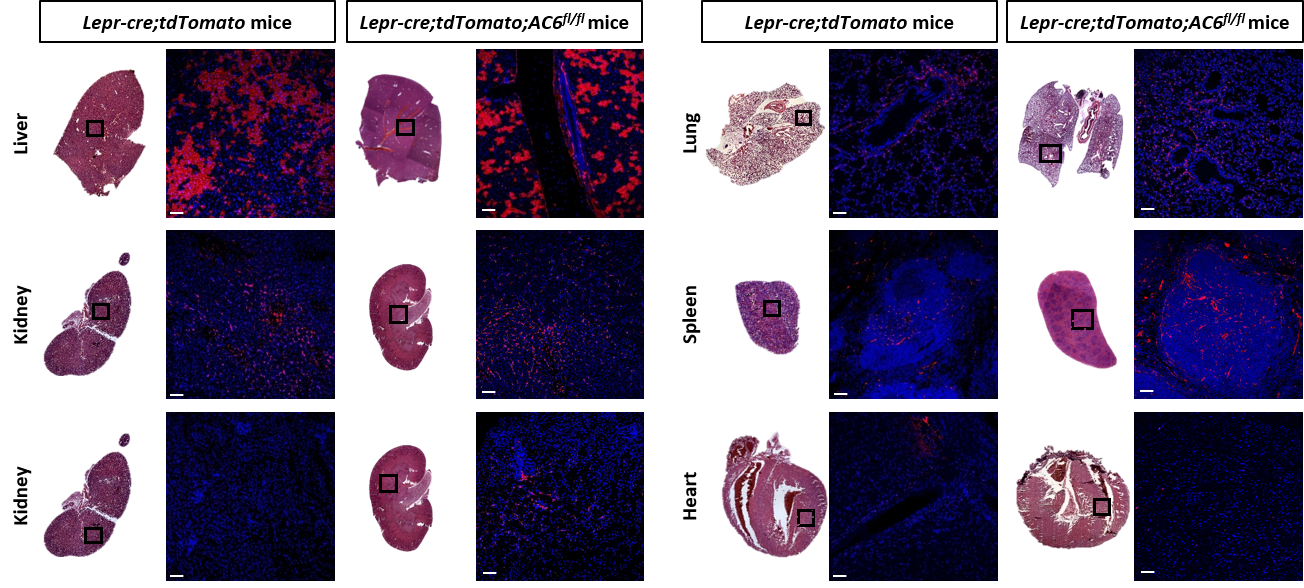
**

**Figure S6:** Patterns of LepR expression in adult *Lepr-cre;tdTomato* and *Lepr-cre;tdTomato;AC6^fl/fl^* mice. To assess whether tdTomato^+^ expression differs with the addition of an Adenylyl cyclase 6 knockdown in adult tissues. Organs were harvested from 14-week-old mice and processed for histological analyses and stained with hematoxylin and eosin or labelled with the nuclear dye DAPI. Scale bar 100µm

**
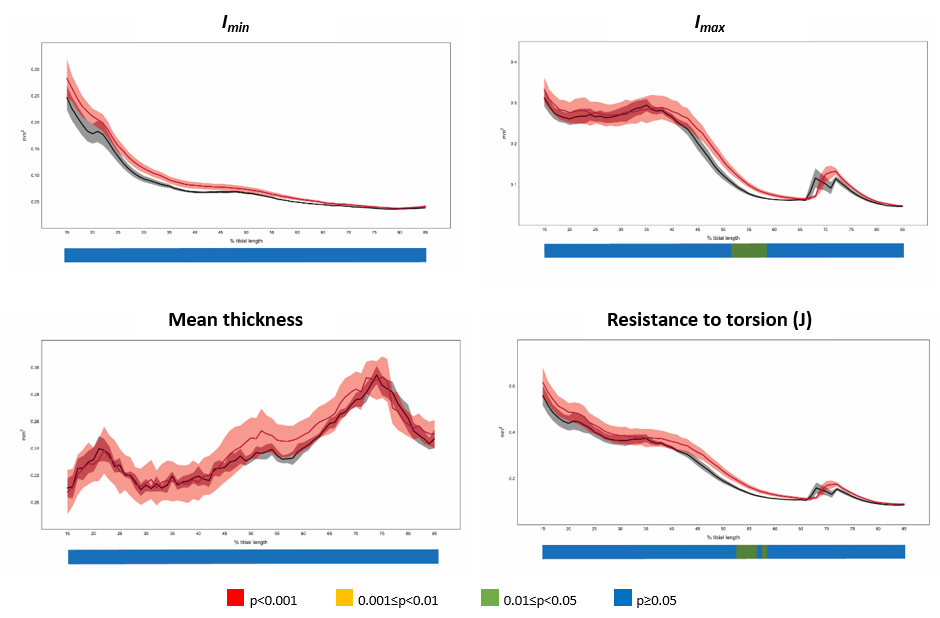
**

**Figure S7:** Axial tibia loading of 12-week-old *Lepr*-cre ;tdTomato^+^;AC6^fl/fl^ mice. Whole bone analyses of cortical bone between 15-90% of the total tibial length, excluding proximal and distal methaphyseal bone showing I_min_, I_max_, mean thickness of the cortex and the resistance to torsion. Loaded: red, static: black, Line graph represent means +/-SEM. Statistical significance of differences along the entire tibial shaft is represented as a heat map, red p< 0.001, yellow 0.001≤p<0.01, green 0.01≤p<0.05 and blue p≥0.05.
